# A peptidoglycan storm caused by β-lactam antibiotic’s action on host microbiota drives *Candida albicans* infection

**DOI:** 10.1038/s41467-021-22845-2

**Published:** 2021-05-07

**Authors:** Chew Teng Tan, Xiaoli Xu, Yuan Qiao, Yue Wang

**Affiliations:** 1 Institute of Molecular and Cell Biology, Agency for Science, Technology and Research, Singapore, Singapore; 2 Department of Biochemistry, Yong Loo Lin School of Medicine, National University of Singapore, Singapore, Singapore; 3 School of Physical and Mathematical Sciences, Nanyang Technological University, Singapore, Singapore

**Keywords:** Antibiotics, Fungal pathogenesis

## Abstract

The commensal fungus *Candida albicans* often causes life-threatening infections in patients who are immunocompromised with high mortality. A prominent but poorly understood risk factor for the *C. albicans* commensal‒pathogen transition is the use of broad-spectrum antibiotics. Here, we report that β-lactam antibiotics cause bacteria to release significant quantities of peptidoglycan fragments that potently induce the invasive hyphal growth of *C. albicans*. We identify several active peptidoglycan subunits, including tracheal cytotoxin, a molecule produced by many Gram-negative bacteria, and fragments purified from the cell wall of Gram-positive *Staphylococcus aureus*. Feeding mice with β-lactam antibiotics causes a peptidoglycan storm that transforms the gut from a niche usually restraining *C. albicans* in the commensal state to promoting invasive growth, leading to systemic dissemination. Our findings reveal a mechanism underlying a significant risk factor for *C. albicans* infection, which could inform clinicians regarding future antibiotic selection to minimize this deadly disease incidence.

## Abstract (web summary)

A risk factor for invasive *Candida albicans* infection is the use of broad-spectrum antibiotics, but the reasons are poorly understood. Here, the authors show in mice that β-lactam antibiotics cause commensal bacteria to release peptidoglycan fragments that induce the invasive hyphal growth of *C. albicans*, leading to systemic dissemination.

## Introduction

*Candida albicans* is an opportunistic fungal pathogen in humans. Although usually a benign member of the human microbiota that inhabits the skin, the oral cavity, the gastrointestinal tract, and the lower female reproductive tract^1,2^, *C. albicans* is also the most prevalent fungal pathogen for nosocomial bloodstream infection with high mortality rates, often exceeding 40%^3^. A major virulence trait of *C. albicans* is its ability to switch between the yeast and hyphal forms of growth^4^. Yeast cells are suited for disseminating in the bloodstream, and hyphae are invasive and can penetrate host mucosal barriers and evade phagocytic cells^2,4^. Although both forms of *C. albicans* are required for virulence, compelling evidence indicates that the commensal state is predominantly the yeast morphology^5–7^. A range of external factors are known to promote hyphal growth, but it remains unclear what triggers the transition from the commensal yeast to invasive hyphae in the host^4^.

Highly adapted to living in the human body, *C. albicans* has an intricate transcriptional regulatory system that responds to diverse signalling molecules released by the host, bacteria and itself to govern the yeast to hyphae morphological switch^4–7^. In the gastrointestinal tract of healthy individuals, *C. albicans* is kept in the yeast state by the combinations of multiple signalling factors, although transient expression of certain hyphae-associated genes is possible. However, genetic or pharmacological perturbations to the host microbiota environment may upset the fine balance to trigger invasive growth of *C. albicans*, leading to candidiasis. What triggers *C. albicans* yeast to hyphal growth in the human body remains unclear although multiple external factors have been implicated^4^. Among known hypha-inducing agents, certain bacterial peptidoglycan (PGN) subunits are extraordinarily active; particularly, 1,6-anhydro-*N*-acetylmuramyl peptides (^anh^MurNAc-pep) exhibit the highest activity^8,9^.

Antibiotics are an essential pillar of modern medicine, which has saved countless lives in the face of deadly bacterial infections^10,11^. However, the enormous scale of overuse and misuse of antibiotics has given rise to new medical problems, creating unprecedented public health threats^12,13^. In addition to the global emergence of antimicrobial resistance^14^, the collateral killing of commensal bacteria by broad-spectrum antibiotics has been associated with significantly increased incidences of many other human diseases, including fatal fungal infections by *C. albicans*^15–17^.

A generally accepted explanation for the correlation between the use of antibiotics and invasive candidiasis is that the killing of commensal bacteria by antibiotics removes competitors, exposes adhesion sites on host epithelial tissues and frees up niches and resources to favour robust fungal proliferation and stable colonization, hence resulting in higher chances of fungal dissemination from the gastrointestinal lumen to visceral organs^15,18,19^. However, this explanation fails to reconcile with recent findings that the depletion of bacteria in the gut does not promote the invasive growth of *C. albicans*. For instance, the antibiotic-facilitated *C. albicans* colonization in the gastrointestinal tract of mice does not result in significant dissemination unless the host is simultaneously immunosuppressed and suffers mucosal damage^20^. Vautier et al. reported that the gastrointestinal tract of mice cleared of bacteria by antibiotic treatment favours the non-invasive yeast growth of *C. albicans*^21^. This observation was further substantiated by a recent report that nearly all *C. albicans* cells found in the gastrointestinal tract of germ-free mice are in the yeast state^5^. There is increasing evidence indicating that the gastrointestinal tract depleted of bacteria by antibiotics strongly selects *C. albicans* mutants defective for hyphal growth^6,22^. Conversely, the gut bacteria may assist *C. albicans* infection under certain circumstances. For instance, *C. albicans* and bacteria commonly coexist at the sites of infection and form mixed-species biofilms^23–26^, and the presence of bacteria indeed increases *C. albicans* virulence and biofilm formation^2,27,28^.

PGN is a conserved component of the bacterial cell wall, which is a meshwork of linear glycan chains composed of alternating β-(1,4)-linked *N*-acetylglucosamine (GlcNAc) and *N*-acetylmuramic acid (MurNAc) cross-linked by regularly spaced short peptides^29^. The monomeric PGN subunit contains one GlcNAc and one MurNAc with a peptide stem attached to MurNAc. To accommodate cell growth and division, bacteria undergo cycles of PGN assembly and disassembly. During disassembly, bacterial lytic enzymes and autolysins hydrolyze PGN polymer to produce various soluble subunits that can be released into the environment^29,30^. Bacterial PGN subunits are known to trigger *C. albicans* hyphal growth by directly binding to the leucine-rich repeat (LRR) domain of the adenyl cyclase in *C. albicans* and activates cAMP synthesis^8,25,31^. cAMP further activates protein kinase A (PKA), leading to the up-regulation of hypha-specific genes responsible for a range of virulence traits, including hyphal growth, adhesion to host tissues, and lysis of host cells^32^. The cAMP–PKA signaling pathway is highly conserved among fungal pathogens and plays a crucial role in pathogenicity^32^.

Most of today’s broad-spectrum antibiotics prescribed in the clinic belong to the β-lactam class^33^, which acts by blocking PGN synthesis^34^. Previous studies suggested that β-lactam treatment causes bacteria to release increased levels of PGN subunits into the culture medium^35^. Recently, Cho et al. reported that β-lactam antibiotics result in a malfunctioning of the PGN synthesis machinery in *Escherichia coli* to churn out large amounts of ^anh^MurNAc-pep molecules^36^. These molecules share a similar structure to the PGN subunits previously shown to be highly active in inducing *C. albcians* hyphal growth^8,36^. These observations led us to hypothesize that β-lactam antibiotic treatment in the host may trigger trillions of commensal bacteria of the gut microbiota to release large quantities of hypha-inducing PGN subunits; this PGN storm would override the hypha-inhibitory factors in host niches to drive *C. albicans* hyphal growth, leading to the penetration of tissue barriers and invasion of internal organs.

In this work, we demonstrate that many bacteria shed PGN subunits that can promote *C. albicans* invasive hyphal growth. The PGN release is markedly increased by treatment with β-lactam antibiotics but not by antibiotics with other mechanisms of action (MOA). Importantly, oral administration of β-lactam antibiotics in mice forces the gut microbiota to release a large amount of hypha-inducing PGN subunits, which transforms the intestinal lumen from a niche that inhibits hyphal growth to one that promotes it, leading to systemic *C. albicans* dissemination. Our findings provide a convincing mechanistic explanation for a significant risk factor of invasive *C. albicans* infections, which imparts valuable information for clinicians on the future use of antibiotics to reduce the incidence of this life-threatening disease.

## Results

### Bacteria release molecules with hypha-inducing activities

**Fig. 1:**
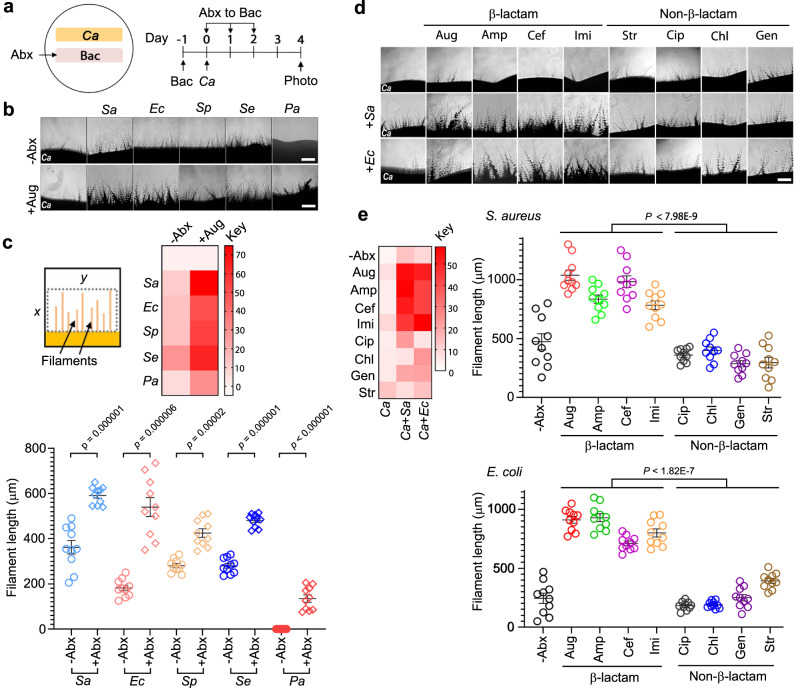
Bacteria promote *C. albicans* hyphal growth and the activity is significantly enhanced by β-lactam antibiotics on plates. **a** Experimental design. 1 × 10^6^ bacterial cells were inoculated into a 0.5 cm × 4 cm area YPD plate and grown at 37 °C for 24 h. Then, *C. albicans* yeast cells were streaked alongside the bacterial patch for incubation at 30 °C for 4 d. See Supplementary Table 1 for all *C. albicans* and bacterial strains used in this study. **b** Microscopic images of *C. albicans* filaments on day 4. Scale bar, 250 µm. The experiment was repeated independently three times with similar results. **c** Quantification of *C. albicans* filamentation shown in (**b**). First, a fixed area of all images was scanned using the mean gray area function in ImageJ. *x* was determined by the average length of ten randomly selected filaments in the image with the strongest filamentous growth, and *y* is the width of the image. The intensity values of all images were normalized against that of *C. albicans* without antibiotic (Abx) treatment and shown by the heatmap (see Methods for detail). Second, 10 filaments (*n* = 10) in each image were randomly chosen to measure the length. *P* values were calculated using two-tailed unpaired *t* test. Error bars = means ± SEM. **d** *C. albicans* was grown alone or side-by-side with *Sa* or *Ec* with or without antibiotic treatment. The results were analyzed as described in (**a**). The antibiotic solution (250 µg for all antibiotics used) was dropped evenly onto the surface of the bacterial patch at 24 h of growth followed by inoculating *C. albicans*. Scale bar, 250 µm. **e** Quantification of filamentous growth as shown in (**c**). Ten filaments (*n* = 10) were measured for each treatment. *P* values were calculated using two-tailed unpaired *t* test. Error bars = means ± SEM.Source data are provided as a Source Data file.

**Fig. 2:**
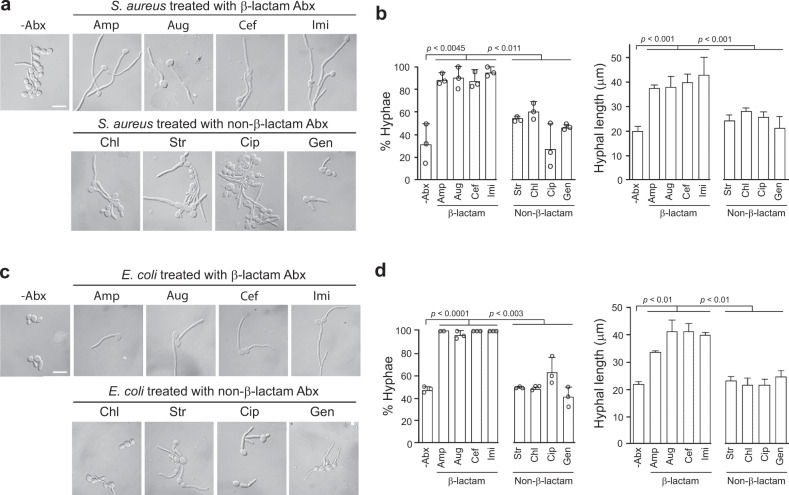
Bacteria promote *C. albicans* hyphal growth and the activity is significantly enhanced by β-lactam antibiotics in liquid cultures. **a**, **c** *Sa* or *Ec* seed cultures were inoculated into fresh LB to OD_595_ = 0.01 and grown at 37 °C with 200 rpm shaking till the cell density reached 10^10^ cells/mL. The culture was split into two parts, and to one was an antibiotic added to a final concentration of 30 µg/mL, which is at least two times the MIC of all antibiotics used on both *Sa* and *Ec* (Supplementary Fig 1a). The cultures were grown for 16 more hours before harvesting the culture supernatant by centrifugation. To test the hypha-inducing activity of the supernatant, *C. albicans* yeast cells were inoculated at 5 × 10^5^ cells/mL into Hank’s Balanced Salt Solution (HBSS) supplemented with 5% of 100-fold diluted bacterial supernatants and incubated at 37 °C for 3 h. Scale bar, 5 µm. The experiment was repeated independently at least three times with similar results. **b**, **d** Percentage and length of hyphae. Results were from three independent experiments. *n* > 50. Scale bar, 250 µm. *P* values were calculated using two-tailed unpaired *t* test. Error bars are means ± SD. Source data are provided as a Source Data file.

To determine whether treating bacteria with β-lactam antibiotics affects *C. albicans* growth state in vitro, we grew *C. albicans* on plates side by side with several common commensal bacteria, including Gram-positive *Staphylococcus aureus* (*Sa*), *Staphylococcus epidermidis* (*Se*), and *Streptococcus pyogenes* (*Sp*) and Gram-negative *Escherichia coli* (*Ec*) and *Pseudomonas aeruginosa* (*Pa*). As described in Fig. 1a, a patch of bacteria was first grown for 1 day. Then *C. albicans* yeast cells were inoculated along the side for co-incubation. Microscopic examination revealed that all bacteria promoted *C. albicans* filamentous growth except for *P. aeruginosa*, which appeared to inhibit filamentation (Fig. 1b, upper row), possibly due to the release of quorum-sensing molecules that inhibit *C. albicans* hyphal germination^23,37,38^. In a pilot test to investigate the β-lactam antibiotic’s effect, we dropped Augmentin (Aug; containing amoxicillin and the β-lactamase inhibitor, clavulanate potassium) solution onto the bacterial patch at 24 h of growth before inoculating *C. albicans* yeast cells. Strikingly, despite killing bacteria, Aug markedly enhanced the hyphal-inducing ability of all bacteria, including *P. aeruginosa*, as evident by the more abundant and longer filaments in the *C. albicans* patch (Fig. 1b, c). Moreover, three other β-lactam antibiotics, ampicillin (Amp), cefoperazone (Cef), and imipenem (Imi) also manifested similar enhancing effects on the *Sa*- and *Ec*-induced filamentous growth of *C. albicans* (Fig. 1d, e). However, on the contrary, antibiotics that do not target bacterial PGN synthesis, such as streptomycin (Str), gentamicin (Gen), chloramphenicol (Chl), and ciprofloxacin (Cip) exhibited no or weak effects on the bacterial*-*induced filamentous growth of *C. albicans* (Fig. 1d, e). None of the antibiotics alone had any effect. Consistently, supernatants of liquid *S. aureus* and *E. coli* cultures treated with β-lactam antibiotics also showed markedly increased hypha-inducing activities (Fig. 2a-d). More than 90% of *C. albicans* yeast cells switched to hyphal growth upon incubation with β-lactam-treated bacterial culture supernatants, whereas <50% of yeast cells became filamentous with the supernatants of untreated or non-β-lactam antibiotic-treated bacterial cultures (Fig. 1b, d, left panel). Furthermore, hyphae induced by β-lactam-treated culture supernatants were about twice as long as the filaments induced with the supernatant of untreated or non-β-lactam antibiotic-treated bacterial cultures (Fig. 2b, d, right panel). Taken together, the results demonstrate that β-lactam antibiotic treatment causes bacteria to release molecules that promote the hyphal growth of *C. albicans*.

### Bacteria-released PGN has potent hypha-inducing activity

**Fig. 3:**
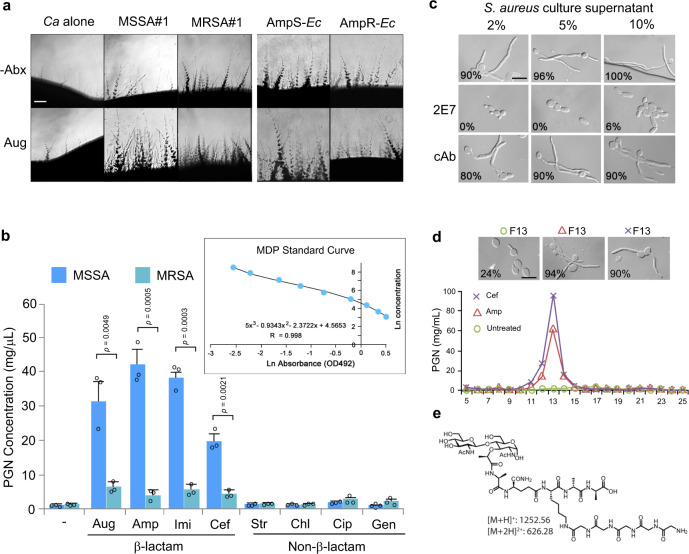
Resistant bacteria release markedly less PGN and hyphae-inducing activity than sensitive strains in response to β-lactam antibiotics. **a** *C. albicans* was grown side-by-side with MSSA#1, MRSA#1, AmpS-Ec, or AmpR-Ec with or without Aug at 30 °C for 4 d. Scale bar, 250 µm. Antibiotic resistance of the resistant strains was confirmed (Supplementary Fig 1b). The experiment was repeated independently three times with similar results. **b** ELISA quantification of PGN in the supernatant of MSSA#1 or MRSA#1 with or without antibiotic treatment. *Sa* supernatnats were prepared as described in Fig. 2a, PGN was quantified by ELISA. Three samples (*n* = 3) for each treatment were analyzed. Authentic MDP was used to prepare the standard curve. Experiments were performed in triplicate. *P* values were determined by two-tailed unpaired *t* test. Source data are provided. **c** Amp-treated *S. aureus* culture supernatant was incubated with 5 mg/mL of 2E7 or a control antibody (cAb) at 37 °C for 1 h before testing the hypha-inducing activity in HBSS at 37 °C for 3 h. Percentage of hyphal growth is shown on each image (*n* = 50). Scale bar, 5 µm. **d** *S. aureus* (MSSA#1) was grown and treated with Cef or Amp, as described in Fig. 2a. In total, 100 mL supernatant was freeze-dried, resuspended into 10 mL sterile water, and fractionated by reversed-phase HPLC. Each fraction was freeze-dried and resuspended into 10 mL sterile water. Then, PGN content in each fraction was quantified by 2E7-ELISA, and the hypha-inducing activity of each fraction was tested at a final concentration of 5% (v/v) in HBSS. Hyphal induction was done at 37 °C for 2 h. Fraction 13 (F13) of both Amp and Cef-treated *S. aureus* cultures have the highest PGN content as well as a high hypha-inducing activity while F13 of untreated culture had low PGN levels and no activity. Fractions outside of the PGN peak areas had no significant activity. Scale bar, 5 µm. The experiment was repeated independently three times with similar results. **e** A muropeptide identified in F13 of Amp and Cef-treated *S. aureus* culture supernatants. Also, see compound e in Supplementary Fig 4.

If the β-lactam antibiotic-triggered bacterial release of PGN subunits is responsible for the enhanced hypha-inducing activity of bacteria, bacterial strains resistant to the drugs would be expected to release less hypha-inducing molecules than the sensitive strains when given the same antibiotic treatment. We compared the hypha-inducing activities of several methicillin-sensitive (MSSA) and resistant (MRSA) *S. aureus* strains side-by-side to test this idea. It was observed that the MSSA strains gave rise to significantly higher levels of *C. albicans* hyphal growth in response to all β-lactam antibiotics tested (Fig. 3a and Supplementary Fig 2). Also, a comparison of sensitive (AmpS-Ec) and resistant (AmpR-Ec) *E. coli* produced similar results (Fig. 3a and Supplementary Fig 2). The AmpR-Ec strain was generated by transforming the sensitive strain with a plasmid that carried an Amp-resistant gene. Antibiotic susceptibilities of the above-mentioned bacteria were verified by disc diffusion assay on plates (Supplementary Fig 1b).

To confirm that bacterial PGN subunits released to the medium are responsible for hyphal growth, we applied our recently developed PGN-targeting monoclonal antibody 2E7 (ref.^39^) that can specifically bind to and neutralize PGN subunits containing the Muramyl-L-Alanine-D-isoGlutamine (MDP) epitope, a highly conserved motif in PGN across all bacteria^40^. Using 2E7-ELISA, we quantified that the MSSA strains released 3**–**8 times more PGN subunits than the MRSA strains when grown in the presence of β-lactam antibiotics under identical growth conditions used in Fig. 2a, while both MSSA and MRSA released more PGN when treated with β-lactam antibiotics as compared to the no treatment controls (Fig. 3b). Furthermore, pre-incubating the *S. aureus* cultural supernatant with 2E7 abolished the hypha-inducing activity, while pre-incubation with a control antibody (cAb) targeting an unrelated epitope did not (Fig. 3c), indicating PGN subunits as the major hypha-inducing factor in the bacterial culture supernatant.

### Identification of hypha-inducing PGN subunits by LC–MS

**Fig. 4:**
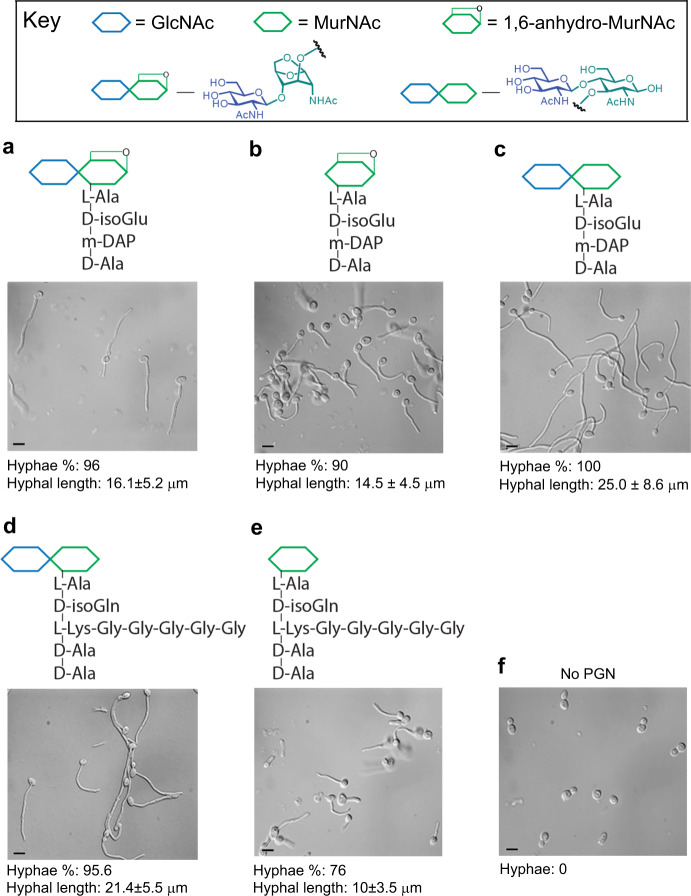
PGN subunits purified from bacterial cell wall have hypha-inducing activity. **a**–**e** PGN subunits . Compound a was purified from *B. pertussis*, and compounds (**a**–**c**) were also isolated from *Ec* cell wall, and compounds (**d**) and (**e**) were purified from *Sa* cell wall. Hypha-inducing activity was tested in HBSS by incubation at 37 °C for 3 h. The concentrations of compounds used for hyphal induction were 500 µM for all compounds. Scale bar, 5 µm. Fifty cells (*n* = 50) were analyzed in each sample, and the hyphal length is mean ± SD. **f** No PGN, negative control. The experiment was repeated independently three times with similar results.

To identify the active PGN subunits, we performed liquid chromatography–mass spectrometry (LC**–**MS) analysis of the HPLC-fractions of β-lactam antibiotic-treated *S. aureus* cultures that contained both high concentrations of PGN (by 2E7-ELISA) and hypha-inducing activity (by hyphal induction test) and identified a prominent muropeptide (Fig. 3d,e; Supplementary Fig 3). This molecule is structurally identical to compound e purified from lysozyme digestion of *S. aureus* PGN polymers (Fig. 4; Supplementary Fig 4a). Together, the results indicate that β-lactam antibiotics cause bacteria to release a large quantity of hypha-inducing PGN fragments.

Although the synthetic compound MDP and the HCl-hydrolysis products of MDP and purified bacterial PGN polymers were previously shown to have hypha-inducing activity on *C. albicans*^8,31^, no naturally released PGN subunits by any bacteria have been found to do so. Tracheal cytotoxin (TCT) is a PGN monomer first purified from *Bordetella pertussis*^40^ and later found to be released by many other Gram-negative bacteria during growth as well^41^. We revealed that TCT indeed exhibits robust hypha-inducing activity (Fig. 4, compound a; Supplementary Fig 4). Furthermore, several PGN fragments purified from enzyme-digested *E. coli* and *S. aureus* PGN polymers also show varying degrees of hypha-inducing potency (Fig. 4, compounds b–e; Supplementary Fig 4). The data demonstrate that many forms of natural bacterial PGN fragments can induce *C. albicans* hyphal growth.

### β-lactam antibiotics promote *C. albicans* hyphal growth in mice

**Fig. 5:**
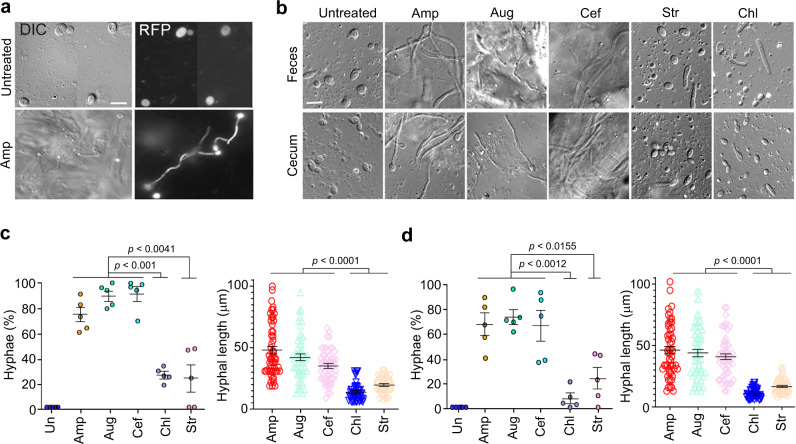
β-lactam antibiotic treatment promotes *C. albicans* hyphal growth in the gastrointestinal tract of mice. **a** *C. albicans* cells in the cecum of mice treated with Amp. DIC, differential interference contrast. All fungal cells found in feces and the intestinal lumen were from the inoculum and endogenous fungal cells were rarely seen, thus the fluorescence images are not shown in panels below. Scale bar, 5 µm. The experiment was repeated independently at least three times with similar results. **b** *C. albicans* hyphae in feces and the cecum of antibiotic-treated and untreated mice. Scale bar, 5 µm. The experiment was repeated independently three times with similar results. **c** Percentage and average length of *C. albicans* hyphae in feces. Five mice were used for each treatment. In total, >50 hyphae were measured for lengths. **d** Percentage and length of *C. albicans* hyphae in the cecum. Five mice were used for each treatment. *P* values were determined using two-tailed unpaired *t* test. Error bars are means ± SEM. Source data are provided as a Source Data file.

**Fig. 6:**
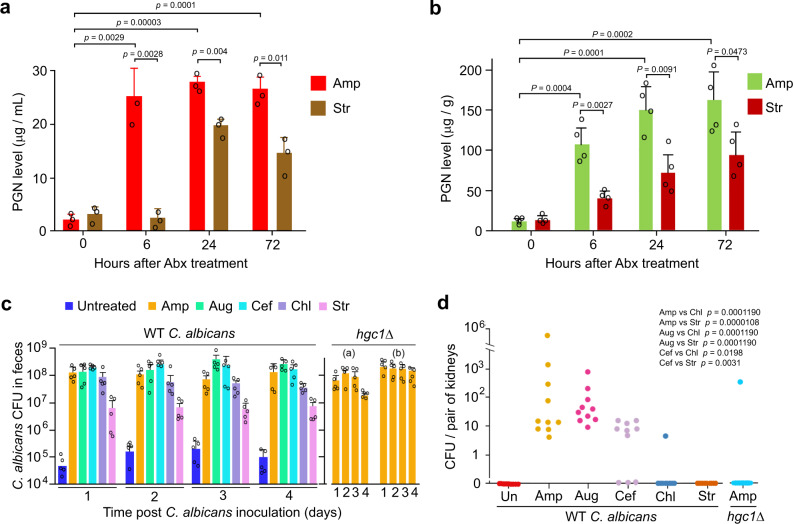
β-lactam antibiotic treatment promotes *C. albicans* dissemination in mice. **a** ELISA quantification of PGN in the lumen of the cecum. Mice (*n* = 3) were sacrificed at the indicated time to harvest the cecum, which was cut open and put into 1 mL of PBS to elute the intestinal content. The eluant was centrifuged at 10,000 *g* for 10 min, and the supernatant was collected for PGN quantification by ELISA. **b** PGN in the feces of mice (*n* = 4) treated with Amp or Str was quantified using the HEK-Blue NOD2 tlr5^-/-^ cell assay. **c** *C. albicans* (WT and *hgc1*Δ/Δ) CFU in the feces of mice (*n* = 5) treated with different antibiotics. *hgc1*Δ (a) and (b), mice were inoculated with 1 × 10^8^ and 2 × 10^8^ *C. albicans* yeast cells, respectively. **d** CFU / kidney of WT *C. albicans* and the *hgc1*Δ/Δ mutant (+*ARG4* +*HIS1* +*URA3*) in the kidney of mice (*n* = 10) treated with different antibiotics. *P* values in (**a**) and (**b**) were determined using two-tailed unpaired *t* test. *P* values in (**d**) were calculated using Fisher’s exact test with odd 95% confidence intervals and alternative = two side in a 2 × 2 contingency table with df = 1. Error bars are means ± SEM. Source data are provided as a Source Data file.

Previous reports indicate that the mouse intestinal lumen restricts *C. albicans* to the yeast state even in the absence of the microbiota^5,6^. We wanted to determine whether oral administration of β-lactam antibiotics can induce *C. albicans* hyphal growth in the gut of mice^20,42^. To minimize the inter-individual variations due to the different drinking behavior of mice, we gavage-fed 7 to 8-week-old Balb/c female mice with the same amount of a β-lactam antibiotic, Amp, Aug or Cef, or a non-β-lactam antibiotic Chl or Str twice on day 1 and also added the same antibiotic in the drinking water throughout the experiment. At 24 h from the start of drug administration, all the antibiotics used reduced bacterial colony forming units (CFU) in feces by 1 × 10^4^–10^5^ fold (Supplementary Fig 5). At this time point, we orally inoculated mice with 1 × 10^8^ wild-type (WT) *C. albicans* yeast cells which express the d-Tomato red fluorescence protein (RFP) for distinction from other fungi in the gut (Fig. 5a). As nearly all the fungal cells found in feces and the gastrointestinal tract were from the inoculated yeast, fluorescent images were not taken in later experiments. At 24 h post *C. albicans* inoculation, we collected fresh feces and sacrificed the mice to harvest the content in the intestinal lumen to examine *C. albicans* morphology. Fig 5a, b show that *C. albicans* cells seen in both feces and the cecum of untreated mice were exclusively in the yeast form, while >70% of *C. albicans* cells in feces and the cecum (also colon) of β-lactam-treated mice were long hyphae. In comparison, in mice treated with a non-β-lactam antibiotic, Str or Chl, 10**–**20% of *C. albicans* cells were filamentous, mostly pseudohyphae with moderate cell elongation, in both feces and the cecum (Fig. 5b-d), and the filaments were also significantly shorter compared to those from mice treated with β-lactam antibiotics (Fig. 5b–d). 2E7-ELISA quantification of PGN in the eluant of the cecal lumen showed a >10-fold increase within 6 h of Amp treatment (Fig. 6a). The marked rise of PGN levels was also confirmed by measuring PGN activation of NOD2 using the HEK-Blue NOD2 tlr5-/- assay system (Fig. 6b). NOD2 is the mammalian intracellular PGN sensor that activates NFκB signaling upon ligand binding^43,44^. We modified HEK-Blue NOD2 cells (Invitrogen) by using CRISPR/cas9 to inactivate TLR5 to prevent the activation of TLR5-NFκB signalling by bacteria-released flagellin present in fecal samples (Supplementary Fig 6). Moreover, feeding mice directly with PGN fragments also led to the observation of significantly increased percentage and length of *C. albicans* hyphal cells in mouse fecal samples, corroborating the hypha-inducing activity of bacterial PGN in the gut (Supplementary Fig 7). These results indicate that treatment with β-lactam antibiotics caused commensal bacteria to flood the intestinal lumen with PGN subunits that dramatically transformed the niche from one that favors the yeast growth to one that promotes robust hyphal growth of *C. albicans*. Although Chl and Str also increased the hyphal growth in the gut, the effect was much less significant than β-lactam antibiotics (Fig. 5c, d). Due to the different MOA, non-β-lactam antibiotics may cause, as a result of cell lysis or death, the release of a smaller amount or certain forms of PGN fragments with no or low hypha-inducing activity^30,39^. We have shown previously that different forms of PGN have drastically different hypha-inducing activities^8^. This could explain why Str-treated mice had a heightened level of PGN in feces, yet the level of *C. albicans* filamentous growth was low and showed no dissemination (Fig. 5c, d and Fig. 6a, b, d).

### β-lactam antibiotics promote *C. albicans* dissemination from the gut

Candidemia in immunocompromised patients is thought to develop from initial gastrointestinal colonization, followed by subsequent translocation into the bloodstream^20,45^. Using mouse models of *C. albicans* gastrointestinal colonization and candidemia, previous studies have demonstrated that both immunosuppression and damages to the gastrointestinal mucosal lining are required for *C. albicans* dissemination^20,46^. Next, we investigated whether the β-lactam antibiotic-induced hyphal growth of *C. albicans* in the gut alone will lead to systemic dissemination from the gastrointestinal tract. The experiment’s design was the same as described above except that after oral inoculation of mice with *C. albicans* yeast cells, fresh feces were collected daily to count *C. albicans* CFU to determine the state of gut colonization. The CFU counts indicated stable *C. albicans* colonization from day 1 to 4 (Fig. 6c). On day 5, we sacrificed the mice to harvest the kidneys to count *C. albicans* CFUs. Strikingly, we found *C. albicans* in the kidney of 26 out of 30 mice that had been treated with a β-lactam antibiotic (Fig. 6d). In stark contrast, no untreated mice (*n* = 10) and only one out of 20 mice treated with a non-β-lactam antibiotic, Chl or Str, had *C. albicans* in the kidney.

It is well established that the ability of *C. albicans* to switch from yeast cells to hyphal growth is central to the pathogenic potential, as hyphal-defective mutants exhibit only very low levels of dissemination from the gastrointestinal tract into extraintestinal organs in antibiotic-exposed mice^20,41^. Next, to confirm that it was the hyphal growth, but not other factors such as drug-induced mucosal damage^47^, responsible for *C. albicans* dissemination, we sought to use hypha-defective mutants to repeat the above experiments. Although several mutants, such as *efg1*Δ/Δ, *ume6*Δ/Δ, *brg1*Δ/Δ, and *tec1*Δ/Δ, are known to be locked in the yeast form, these genes all encode transcription factors that control the expression of multiple virulence-related traits besides hyphal growth^22,48–50^. The use of these mutants could mislead the conclusion on the role of hyphal growth^22,51^. Thus, we selected the *hgc1*Δ/Δ mutant for the experiment as *HGC1* is one of the numerous hyphal-specific genes co-regulated by the above transcription factors and plays a specific role in regulating hyphal morphogenesis^52^. Repeating the gut colonization-dissemination experiment in β-lactam antibiotic-treated mice using the *hgc1*Δ/Δ mutant, we detected *C. albicans* cells in the kidney of only one out of ten mice despite that *hgc1*Δ/Δ cells colonized the gut to a level comparable to the WT strain (Fig. 6c, d). Also, the *hgc1*Δ/Δ mutant does not have a compromised ability to colonize the kidney, as it manifested comparable CFUs in the kidney to the WT *C. albicans* following tail-vein injection (Supplementary Fig 8). Thus, the rare detection of *hgc1*Δ/Δ CFUs in mouse kidneys in the gut colonization-dissemination experiment indicates that the hyphal growth of *C. albicans* is the cause of its dissemination from the gut. Taken together, these results strongly support the idea that the β-lactam antibiotic-triggered PGN release and the subsequent activation of hyphal growth markedly increase *C. albicans* dissemination from the gastrointestinal tract. However, we cannot exclude the possibility that some bacteria that survived the antibiotic treatment might help *C. albicans* dissemination.

## Discussion

**Fig. 7:**
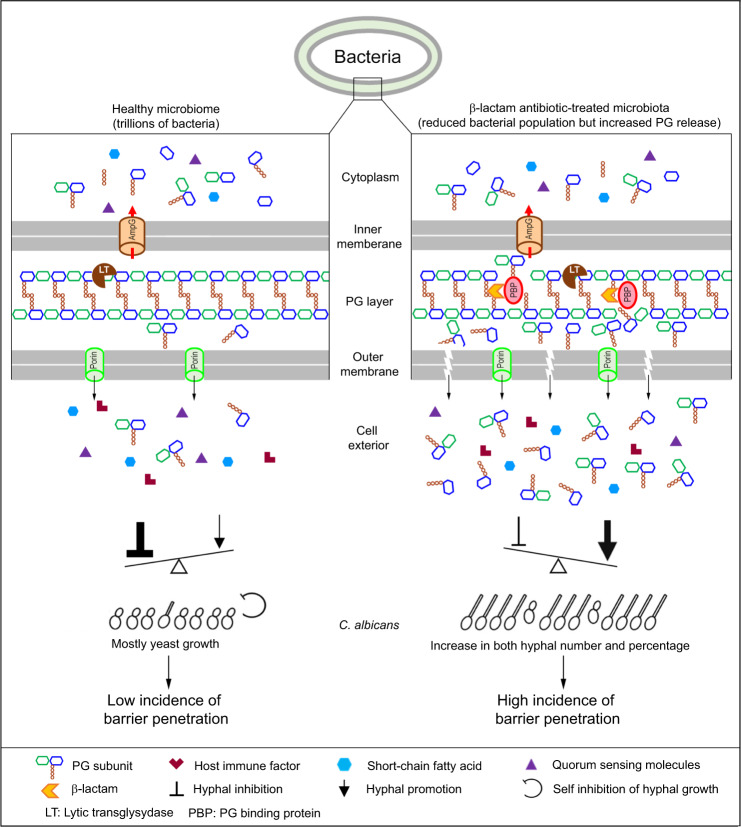
A model depicting the mechanism by which β-lactam antibiotic treatment increases the risk of invasive *C. albicans* infection. The left column depicts the microbiota-*C. albicans* interactions in the gastrointestinal tract of healthy individuals, where the hyphal growth of *C. albicans* is kept in check by the combined actions of different classes of inhibitory molecules including microbiota-derived quorum-sensing molecules and short-chain fatty acids, host-derived antimicrobial peptides and immune effectors, as well as the self-repression system of *C. albicans*. There is a low incidence of *C. albicans* hyphal growth and mucosal barrier penetration for dissemination. The right column depicts the effects of β-lactam antibiotics on human gut microbiota that tip the balance in favor of *C. albicans* hyphal growth. β-lactam antibiotics inhibit the bacterial PGN assembly, which causes the generation of hyphal-inducing PGN subunits and lysis of bacteria cells, resulting in the release of a massive amount of PGN subunits. The β-lactam antibiotics-induced PGN storm transforms the gastrointestinal environment from inhibitory to favorable to the hyphal growth of *C. albicans*, thus vastly increasing the number of hyphae and the probability of breaching the mucosal barrier for dissemination. For the structure and components of the bacterial cell wall, proteins involved in PGN recycling, and the target and MOA of β-lactam antibiotics, please refer to previous publications^29,30^. The figure was prepared by YQ and YW.

Understanding the mechanisms behind risk factors is critical for the prevention and management of a disease. In addition to a compromised immune system and damages to mucosal barriers, another well-recognized risk factor for invasive candidiasis is the use of broad-spectrum antibiotics, although the underlying mechanisms remain unclear. Numerous previous studies have shown that the host gastrointestinal microbiota plays a vital role in preventing *C. albicans* colonization and dissemination. Investigators have offered a range of explanations for the host microbiota’s protective function, including blocking *C. albicans* mucosal association, releasing molecules that inhibit either *C. albicans* growth or hyphal formation, and competition for essential nutrients^3,13,15,22,53^. These ideas naturally lead to the concept that treatment with antibiotics removes or weakens these protective factors and consequently increases fungal infection. While these factors may all contribute to the increased risks of invasive *C. albicans* infection as well as infections by other fungal pathogens, our discoveries in this study reveal another mechanism that, we believe, plays the most crucial role in promoting *C. albicans* dissemination following treatment with β-lactam antibiotics, the most frequently prescribed class of antibiotics^54^. We propose the following model (Fig. 7). In the gastrointestinal tract of healthy people, several factors restrain *C. albicans* mainly in the commensal yeast state, thus preventing infection. First, commensal bacteria vastly outnumber *C. albicans* and release various hyphal inhibitory molecules, such as quorum-sensing molecules and short-chain fatty acids^55–57^. Second, there are host-derived hyphal inhibitors, possibly some antimicrobial peptides and other immune effectors^5,21^. Third, *C. albicans* may actively repress the hyphal program through a specific transcriptional program to maintain its fitness as hyphae may be detrimental to its survival in the gastrointestinal tract^5,6,58^. These negative regulatory factors of hyphal growth prevail over hypha-inducing factors, such as bacteria-released PGN and body temperature. However, treatment with β-lactam antibiotics will cause *C. albicans* overgrowth together with the release of a massive amount of hypha-inducing PGN subunits by the trillions of gut bacteria via increased generation of PGN subunits and eventual autolysis of cells, which transforms the gastrointestinal environment from favoring yeast growth to promoting hyphal growth, thus vastly increasing the number of *C. albicans* hyphal cells and the incidence of penetrating the mucosal barrier. Guinan et al. recently reported that treatment of mice with cefoperazone, a β-lactam antibiotic, increased the level of taurocholic acid (TCA) and decreased that of short-chain fatty acids (SCFAs) in the gastrointestinal tract^59,60^, which could also contribute to the increase of the hyphal growth of *C. albicans* we observed. However, whether other antibiotics can also cause similar changes in TAC and SCFA levels to the gastrointestinal commensal bacteria requires further investigation. It should be mentioned that while the gut environment strongly favors yeast growth, there are niches that may allow transient hyphal growth. Perez et al. identified eight transcription factors that control genes required for gastrointestinal tract colonization, systemic infection, or both^61^. These transcription factors form a dynamic regulatory circuit highly responsive to environmental cues. We propose that the β-lactam antibiotics-induced PGN storm in the gut tips the balance towards invasive hyphal growth.

Using ELISA and NOD2 activity assays, which detect PGN by independent mechanisms, we observed a sharp increase in the gut PGN level following oral β-lactam antibiotic administration in mice. Intriguingly, the gut PGN level remained high for days. This phenomenon is likely due to the persistence and growth of antibiotic-resistant bacteria that continue to release PGN subunits in the drug’s presence. Ng et al.^62^ recently demonstrated that while feeding mice with antibiotics caused a precipitous drop in the total number of gut bacteria initially, many species recovered to the pretreatment or a higher level within as few as one day despite continued drug administration. Similar recovery was observed after treatment with six antibiotics with different MOA, including streptomycin, ciprofloxacin, amoxicillin, clindamycin, metronidazole, and rifaximin^62^. Consistently, we also observed the recovery of aerobic bacteria to ~50–60% of the pretreatment level at 72 and 96 h of Amp treatment (Supplementary Fig 9). In Fig. 3, we show that while resistant bacteria release significantly less amount of PGN than sensitive ones in response to β-lactam antibiotics, they still released more PGN in the presence of the drug than the untreated controls. Thus, the continued PGN release from resistant bacteria at elevated levels during β-lactam antibiotic treatment provides a reasonable explanation for the persistently high PGN levels in the gut.

Murine models of *C. albicans* gastrointestinal colonization and dissemination have been used extensively to simulate one major route of systemic *C. albicans* infection in humans^63^. However, *C. albicans* is not a natural commensal or pathogen in mice^64^. Adult mice are naturally resistant to *C. albicans* gastrointestinal colonization^20,65^. For stable colonization of *C. albicans* in the gastrointestinal tract, pretreatment of mice with oral antibiotics before *C. albicans* inoculation is required, which creates an unnatural gastrointestinal environment by removing the commensal bacteria. Thus, great caution should be taken in explaining *C. albicans* behavior observed using this model. When we inoculated mice with *C. albicans* yeast cells via oral gavage without pre-antibiotic treatment, nearly all *C. albicans* cells detected along the entire gastrointestinal tract or in feces 6–48 h post inoculation were in the yeast form, indicating that the normal gastrointestinal lumen of mice inhibits *C. albicans* hyphal growth. Abundant hyphal growth was detected only when mice were pretreated with a β-lactam antibiotic. However, lower levels of filamentous growth were also observed after treatment with a non-β-lactam antibiotic such as Chl or Str. We noted significant discrepancies in the descriptions of *C. albicans* morphologies in the gastrointestinal tract of mice in previous reports. While Witchley et al.^22^ reported that *C. albicans* colonized the gastrointestinal tract as a mixture of yeast and hyphae in mice that had been given the drinking water containing penicillin (1500 µ/mL), Str (2 mg/mL), and glucose for 7–8 days, Vautier et al.^21^ observed that *C. albicans* gastrointestinal colonization primarily favored yeast cells in mice that had been provided with the antibiotic water containing penicillin (2000 µ/mL), Str (2 mg/mL), and fluconazole (0.25 g/mL) before *C. albicans* inoculation. We also detected only yeast cells in the feces of mice treated with the mixture of penicillin (2000 µ/mL) and streptomycin (2 mg/mL) (Supplementary Fig 10). It is plausible that on the one hand, the antibiotic treatment removed bacteria that produced inhibitory molecules to favor *C. albicans* hyphal growth. On the other hand, penicillin or other β-lactam antibiotics in the drinking water caused gastrointestinal bacteria to release hypha-inducing PGN subunits as we demonstrated in this study. The balance of the two opposing effects and the significant variations between protocols (dosage, duration of treatment, types of antibiotic used, means of antibiotic administration, etc.) could influence the ratio between yeast and filamentous cells and explain the discrepancies in results reported by different laboratories. In support of the inhibitory effect of the gastrointestinal tract on *C. albicans* hyphal growth, Böhm et al. recently reported that *C. albicans* cells found in the gastrointestinal tract of germ-free mice (in the absence of antibiotic treatment) almost uniformly adopted the yeast cell form^5^.

To clear the gut of bacteria in mice, all previous studies used antibiotic cocktails containing one β-lactam antibiotic, often penicillin, combined with other antibiotics with different MOA. The lack of significant hyphal growth of *C. albicans* in the gut in these studies could be due to multiple factors: (1) Antibiotic cocktails kill bacteria rapidly via synergistic effect, leaving little time for the hyphal-inducing PGN subunits to accumulate than when treated with a β-lactam antibiotic alone. (2) Significantly fewer bacteria can survive when exposed to two or more antibiotics with different MOA. (3) Combinations of antibiotics may result in gut bacteria releasing a mixture of both hypha-inducing and inhibiting metabolites, which may obscure the effect of PGN. (4) Antibiotics that inhibit protein synthesis are antagonistic to the mode of action of β-lactams and therefore can block the lysis characteristic of β-lactams. Furthermore, differences in antibiotic administration protocols also contribute to the observed discrepancies in *C. albicans* growth morphology in previous studies. Our study used a single antibiotic, either β-lactam or non-β-lactam, which allowed us to delineate each drug’s specific effect on the gut microbiota. Our results strongly support the idea that the β-lactam treatment triggers a PGN storm in the gut that in turn promotes *C. albicans* hyphal growth and dissemination.

Our findings open up possibilities for designing new strategies to reduce the risk of life-threatening *C. albicans* invasive infections in immunocompromised patients by modifying the selection and regimen of antibiotic therapies in the future. As bacterial PGN subunits are immune regulators^39,44^ and have been causally linked to several diseases^66–68^, the β-lactam antibiotic-induced PGN storm has a much broader impact on human physiology and health.

## Methods

### Growth conditions of *C. albicans* and bacteria

*C. albicans* was routinely grown in YPD (1% yeast extract, 2% peptone, and 2% glucose) at 30 °C and bacteria in LB media (Sigma Aldrich) at 37 °C. Both *C. albicans* and bacteria liquid cultures were grown with shaking at 200 rpm. To prepare the bacterial culture supernatant, a seed culture was diluted to OD_595_ = 0.01 with fresh LB medium and incubated at 37 °C with 200 rpm shaking for ~8 h until the cell density reached 10^10^ cells/mL. After adding an antibiotic to a final concentration of 30 µg/mL, the incubation was continued for another 16 h. The culture supernatant was harvested after centrifugation at 5000 g for 10 min.

### Bacterial induction of *C. albicans* hyphal growth on plates

Approximately 11 × 10^6^ bacterial cells were inoculated into a 0.5 cm × 4 cm area on the surface of YPD agar plates and incubated at 37 °C for 24 h. Then, *C. albicans* yeast cells were streaked along the side of the bacterial patch, and incubation was continued at 30 °C. The growth morphology of *C. albicans* cells was examined daily under a microscope. Bacterial strains used in the experiments are shown in Supplementary Table 1. To determine the effect of antibiotics on the bacteria-induced hyphal growth of *C. albicans*, an aliquot of antibiotic solution (containing 250 µg for all antibiotics used) was dropped evenly on the surface of the bacterial patch.

### *C. albicans* hyphal induction in vitro

*C. albicans* hyphal growth was induced in Hank’s Balanced Salt Solution (HBSS; Sigma-Aldrich) containing 5 × 10^5^ overnight yeast cells and incubated at 37 °C for 2–3 h.

### Preparation of bacterial muropeptides

For the experiments shown in Fig. 3c, d, a 100 mL aliquot of β-lactam antibiotic-treated *S. aureus* culture broth were freeze-dried, resuspended into 10 mL sterile water, and fractionated by reversed-phase HPLC. Each fraction was freeze-dried and resuspended into 10 mL sterile water. Then, PGN content in each fraction was quantified by 2E7-ELISA, and the hypha-inducing activity of each fraction was tested at a final concentration of 5% (v/v) in HBSS and 37 °C for 2 h.

Muropeptides shown in Fig. 4 were isolated by high-performance liquid chromatography (HPLC) from lysozyme digestion of PGN polymers isolated from *Staphylococcus aureus* and *Escherichia coli*, which were prepared according to published protocols^69^. For the preparation of 1,6-anhydro MurNAc form of muropeptides, PGN polymers were digested with membrane-bound lytic transglycosylase B (MltB) from *E. coli*^70^. TCT was also provided by William E. Goldman, who purified it from *Bordetella pertussis*^71^. *E. coli* MltB was cloned into pET21b as a C-terminally His-tagged protein, where the first 21 amino acids were truncated. For the preparation of monosaccharide MurNAc muropeptides, the corresponding disaccharide muropeptides were digested with *Pseudomonas aeruginosa* NagZ, which encodes the β-hexosaminidase that cleaves off the GlcNAc residue in disaccharide muropeptides. PAO_NagZ was cloned into pET28a as an N-terminal His-tagged protein with the first 27 amino acid deleted, and purified as previously reported^72^. HPLC purified desired muropeptides, and their structures were confirmed by mass spectrometry. High resolution MS2 data was obtained by Vanquish Core Binary HPLC coupled with Orbitrap Exploris 120 instrument.

### ELISA of PGN

Reagents used in the ELISA for PGN quantification include 2E7 (ref.^39^), human serum albumin (HSA)-MDP complexes, and horseradish peroxidase (HRP)-conjugated anti-mouse antibody (GE Health), MDP (Sigma-Aldrich), o-phenylenediamine dihydrochloride (OPD) (Acros Organics), hydrogen peroxide (VWR international), and 4 M sulfuric acid. The following buffers or solutions were used. Coating buffer: 1.59 g of Na_2_CO_3_ and 2.93 g of NaHCO_3_ were dissolved in 1 L of distilled water, and the pH was adjusted to 9.5; coating solution: stock MDP-HSA was diluted in the coating buffer to the working concentration of 2 µg/mL; blocking buffer: 0.1 g of gelatin was dissolved in 1 L of the coating buffer; washing buffer: 5 mL of 20% Tween-20 was dissolved in 2 L of PBS; antibody buffer: 0.1 g of gelatin was dissolved in 1 L of the washing buffer; substrate buffer: 19.2 g of citric acid and 28.4 g of Na_2_HPO_4_ were dissolved in 1 L of distilled water and the pH was adjusted to 5; 2E7 solution: 1 mg/mL stock was diluted 1:4000 in 21 × antibody buffer; secondary antibody solution: anti-mouse IgG antibody with HRP conjugation (GE Health, USA) was diluted 1:2000 with the antibody buffer; and OPD substrate solution: 5 mg of solid OPD was dissolved in 10 mL of the substrate buffer, and 0.32 µL/mL hydrogen peroxide (VWR international) was added before use. ELISA was performed in ½-area 96-well plates (Costar®, Corning, USA). Then, 50 µL of the coating solution was added into each well, and the plate was incubated at 4 °C overnight. The plates were blotted dry, 50 µL of the blocking buffer was added to each well, and the plate was incubated at 37 °C for 1 h. Then, the wells were washed using the washing buffer three times and blotted dry. The 2E7 solution was mixed with the sample (such as bacterial culture supernatant) at 1:1 ratio and incubated at room temperature for 1 h. Then, 50 µL of the mixture was added into each well of the coated plate and incubated at 37 °C for 1 h. The plate was then washed three times, each with 100 µL of the washing buffer, and blotted dry after each wash. Then, 50 µL of the secondary antibody was added to each well and incubated at 37 °C for 1 h followed by washing four times, each with 100 µL of the washing buffer. Then, 50 µL of the OPD substrate solution was added to each well and incubated for 2–5 min at 37 °C in the dark for color development. To terminate the reaction, 25 µL of 4 M sulfuric acid was added to each well. Absorbance was read at 492 nm wavelength using a microtiter plate reader (Tecan infinity Pro200). The PGN level in a sample is inversely correlated with the optical density of the reaction products. A standard curve was generated using 2-fold serial dilutions of a known MDP concentration each time when the assay was performed.

### PGN assay using HEK-Blue NOD2 and NOD2 tlr5-/- cells

HEK-Blue NOD2 cells were purchased from InvivoGen. Mouse fecal samples also contain bacterial flagellin that activates NF-κB signalling via TLR5. To specifically assay the PGN activation of NF-κB signalling via NOD2, we used CRISPR-cas9 to inactivate TLR5 in HEK-Blue NOD2 cells, yielding the HEK-Blue NOD2 tlr5-/- strain for quantifying PGN in fecal samples. The nucleotide sequence of gRNA used to inactivate TLR5 (n.t.69-91) by CRISPR is 5ʹ-CCTGCTCCTTTGATGGCCGAATA-3ʹ. CRISPR inactivation of TLR5 in HEK293 NOD2 cells was conducted following established protocols^73^. DNA sequencing showed that the CRISPR-induced mutation caused a frameshift, creating a premature stop codon at amino acid 61. The mutation completely blocked the cellular response to flagellin treatment (Supplementary Fig 6). The MDP/PGN assay was performed following the provider’s instruction. The growth medium used was Dulbecco’s Modified Eagle Medium (DMEM)/High Glucose supplemented with penicillin (100 µg/mL), streptomycin (100 µg/mL), normocin^TM^ (100 µg/mL), zeocin^TM^ (100 µg/mL), blasticidin (30 µg), and 10% fetal bovine serum. Vials of HEK-Blue NOD2 or HEK-Blue NOD2 tlr5-/- cells taken from a liquid nitrogen tank were thawed with gentle agitation in a 37 °C water bath and then transferred to 10 mL of prewarmed growth medium. Cells were sedimented by centrifugation at 150 g 4 °C for 5 min. The supernatant was discarded, and the cells were resuspended in 10 mL prewarmed growth medium and transferred to a 10 × 1.5 cm cell culture plate for incubation in 5% CO_2_ at 37 °C. When the culture grew to ~80% confluent, the cells were transferred to fresh medium and placed in 5% CO_2_ at 37 °C. After two passages, the cells were resuspended in growth medium to a concentration of 2.8 × 10^5^/mL. For MDP assay, 20 µL of a sample (serum or standard MDP) was added to each well of a flat-bottom 96-well plate, followed by 180 µL of HEK Blue NOD2 or HEK Blue NOD2 tlr5-/- cells and gentle mixing. The plates were incubated in 5% CO_2_ at 37 °C for 12–16 h. Then, 10 µL of the supernatant from each well was transferred to a well of a 96-well half-area plate and then mixed with 90 µL QUANTI-Blue solution (InvivoGen). The plate was incubated at 37 °C for 30 min, and optical density at 655 nm was measured using a plate reader. Two-fold serially diluted standard MDP was included to generate the standard curve.

### Fractionation of soluble PGN in *S. aureus* culture supernatants

Bacterial culture supernatants were prepared as described above and subjected to HPLC fractionation using a reversed-phase C18 column (Atlantis dC18 19 mm × 100 mm column) from Waters. Solvent A was water and solvent B acetonitrile, both containing 0.01% trifluoroacetic acid (TFA). A linear solvent gradient was used: 0% B, 0–1 min with the flow rate increased from 0 to 10 mL/min; 0–40% B, 1–61 min; 40–0% B, 61–66 min; 0% B, 66–76 min. Fractions were collected at 1-min intervals, lyophilized, and re-suspended in sterile water for ELISA quantification of PGN. The fractions containing PGN were then subjected to LCMS analysis using a Thermo Accela LCQ Fleet LC–MS equipped with a C18 column (Waters symmetry shield RP18, 3.9 mm × 150 mm). The following gradient was used for the analysis: 100% A (Water + 0.1% formic acid) for 0–5 min, followed with a linear gradient of 0–15% B (acetonitrile + 0.1% formic acid) for 5–20 min. The mass corresponding to the known PGN fragment was identified.

### Quantification of filamentous growth on plates

First, the area of an image occupied by filamentous growth was measured using the mean gray area (MGA) function in ImageJ. *C. albicans* alone without antibiotic treatment which has the least filamentous growth will always give a bigger MGA value (V0) than others value (V1). A value (Vf) that represents the degree of filamentous growth in each image was calculated using the formulae V0 − V1 = Vf, where Vf greater than zero represents an enhancement of filamentous growth, and Vf was presented in a heat map. Occasionally, V0 is smaller than V1, and the Vf becomes a negative value, which was considered as no enhancement of hyphal growth.

### Animal experiments

Female Balb/c mice aged 7–8 weeks (purchased from InVivos, Singapore) and five mice were kept in a cage. Mice were housed in SPF conditions. All animal rooms had a controlled environment with 12/12 light/dark cycles, 20–22 °C temperature, 0.3 micron HEPA (high-efficiency particulate air) filtered air supply at 15–20 ACPH, and 50–60% relative humidity. All mice were administered orally with 0.5 mL of an antibiotic solution (8 mg/mL for all antibiotics used except for Cef which was at 6 mg/mL) twice per day on day 1 and also given drinking water supplemented with the same antibiotics at 4 mg/mL throughout the experiment. The untreated mice were treated exactly the same way as the antibiotic-treated mice except that they were given pure water instead of antibiotic-containing water. At 24 h of the antibiotic treatment, mice were orally inoculated with 1 × 10^8^ *C. albicans* yeast cells, and feces were collected at intervals to either count CFU or examine *C. albicans* morphology. Fresh stool pellets were collected in the morning by gently pressing the abdomen of mice. Feces were weighed, resuspended in ice-cold sterile PBS, homogenized, serially diluted, and spread onto LB agar plates to count bacterial CFU to determine the effectiveness of antibiotic treatments in reducing the number of gut bacteria. In some experiments, mice were sacrificed at a time point after inoculation with *C. albicans* yeast to harvest the intestine for microscopic examination of *C. albicans* morphology and analysis of PGN content in the lumen. To determine *C. albicans* systemic dissemination from the gastrointestinal tract, mice were killed 5 days post inoculation with *C. albicans* yeast cells to harvest the kidney to count CFU. Mice were randomly assigned to control and treatment groups. Reproducibility was confirmed by multiple independent experiments. All animal experiments were performed following animal ethics guidelines and protocols approved by the Institutional Animal Care and Use Committee (IACUC) of the Agency for Science, Technology and Research of Singapore.

### Microscopy

A Leica inverted DM Rb microscope attached with a Moticam Camera interfaced with Motic Images 2.0 software was used for the imaging of *C. albicans* filaments on plates. A Leica DMR microscope fitted with a Hamamatsu digital camera interfaced with Metamorph software was used to take microscopic images of *C. albicans* cells. For the d-Tomato red fluorescent signal, the rhodamine filter was used. All images were processed using Adobe Photoshop.

### Statistics and reproducibility

Graph Pad Prism Software Version 4.00 and R were used for all statistical analyses. Results of ELISA, NOD2 assays, and hyphal induction were analyzed by two-tailed unpaired *t* test. For the animal experiment shown in Fig. 6d, the data were analyzed using Fisher’s exact test. All experiments were repeated independently at least three times with similar results.

### Reporting summary

Further information on research design is available in the Nature Research Reporting Summary linked to this article.

## Supplementary information

Supplementary Information

Reporting Summary

## Data Availability

All data that support the findings of this study are either included in this published article and its Supplementary information or available from the corresponding author upon request. Source data are provided with this paper.

## Source data

Source Data
