# Author Correction: A peptidoglycan storm caused by β-lactam antibiotic’s action on host microbiota drives *Candida albicans* infection

**DOI:** 10.1038/s41467-021-23868-5

**Published:** 2021-06-03

**Authors:** Chew Teng Tan, Xiaoli Xu, Yuan Qiao, Yue Wang

**Affiliations:** 1 Institute of Molecular and Cell Biology, Agency for Science, Technology and Research, Singapore, Singapore; 2 Department of Biochemistry, Yong Loo Lin School of Medicine, National University of Singapore, Singapore, Singapore; 3 School of Physical and Mathematical Sciences, Nanyang Technological University, Singapore, Singapore

**Keywords:** Antibiotics, Fungal pathogenesis

Correction to: *Nature Communications* 10.1038/s41467-021-22845-2, published online 7 May 2021.

The original version of this Article omitted the following from the Acknowledgements:

Y.Q. was supported by the National Research Foundation (NRF) of Singapore, NRF-NRFF12-2020-0006.

This has now been corrected in both the PDF and HTML versions of the Article.

