# Supplementary material for: A peptidoglycan storm caused by β-lactam antibiotic’s action on host microbiota drives Candida albicans infection
Source: Nat Commun. 2021 May 7;12:2560. doi: 10.1038/s41467-021-22845-2 (PMC8105390; doi:10.1038/s41467-021-22845-2)
Supplement: Supplementary file 1 — Supplementary Information [file 41467_2021_22845_MOESM1_ESM.pdf]

## Supplementary Information

### A peptidoglycan storm caused by β-lactam antibiotics' action on host microbiota drives *Candida albicans* infection

Tan et al.

Supplementary Figure 1. Susceptibility of *S. aureus* and *E. coli* strains to various antibiotics.

Supplementary Figure 2. Resistant bacteria release significantly less PGN and hyphae-inducing activity than sensitive strains in response to β-lactam antibiotics.

Supplementary Figure 3. Identification of a muropeptide in the supernatant *S. aureus* culture treated with Cef.

Supplementary Figure 4. HPLC purification, MS identification, and hyphal induction activity of the six muropeptides described in Figure 3.

Supplementary Figure 5. Test of the effectiveness of antibiotics on reducing the number of bacteria in the gut.

Supplementary Figure 6. HEK293 NOD2 tlr5-/- cells do not respond to flagellin while respond to MDP normally.

Supplementary Figure 7. Oral administration of mice with PGN caused *C. albicans* hyphal growth in the gut.

Supplementary Figure 8. WT *C. albicans* and the *hgc1*Δ/Δ mutant showed comparable ability to colonize mouse kidneys.

Supplementary Figure 9. Recovery of bacterial growth during Amp treatment.

Supplementary Figure 10. Morphology of *C. albicans* in the feces of mice treated with the mixture of penicillin and streptomycin.

Supplementary Table 1. *C. albicans* and bacterial strains used in this study.

Supplementary Figure 1

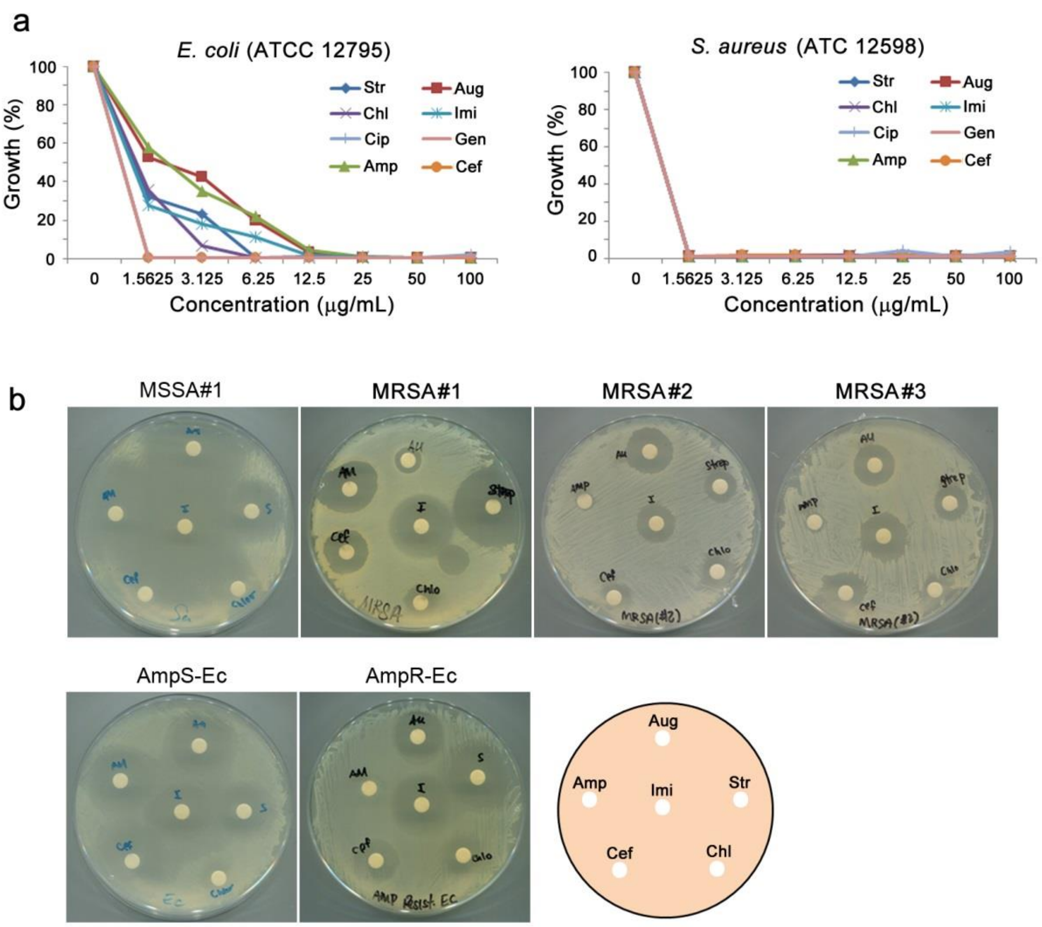


**Supplementary Figure 1**. Susceptibility of *S. aureus* and *E. coli* strain to various antibiotics.

**a** Disc diffusion assay of sensitive and resistant *S. aureus* and *E. coli* strains on LB plates. Bacteria ($1 \times 10^8$ cells) were evenly spread on the plate. Then, paper discs loaded with 250 µg (for sensitive strains) or 500 µg (for resistant strains) of the indicated antibiotics were placed onto the surface. The plates were incubated at 37°C for 24 h. MSSA#1, ATCC12598; MRSA#1, BAA1769; MRSA#2 and #3 are clinical isolates that were characterized and provided by the Infectious Disease Division, National University Hospital of Singapore. (Supplementary Table 1). AmpS-Ec, XL-1-Blue *E. coli*; AmpR-Ec, XL-1-Blue *E. coli* transformed with a plasmid carrying an AmpR gene.

**b** Minimum inhibitory concentration (MIC) assay. Bacteria cells ($1 \times 10^6$ cells/mL) were inoculated into 200 µL of LB medium containing 2-fold serially-diluted antibiotics in wells of a 96-well microtiter plate and incubated at 37°C for 24 h. Bacterial growth in each well was measured using a microtiter plate reader at 595-nm wavelength. Bacterial growth in the well without antibiotic was treated as 100% growth.

Suplementary Figure 2

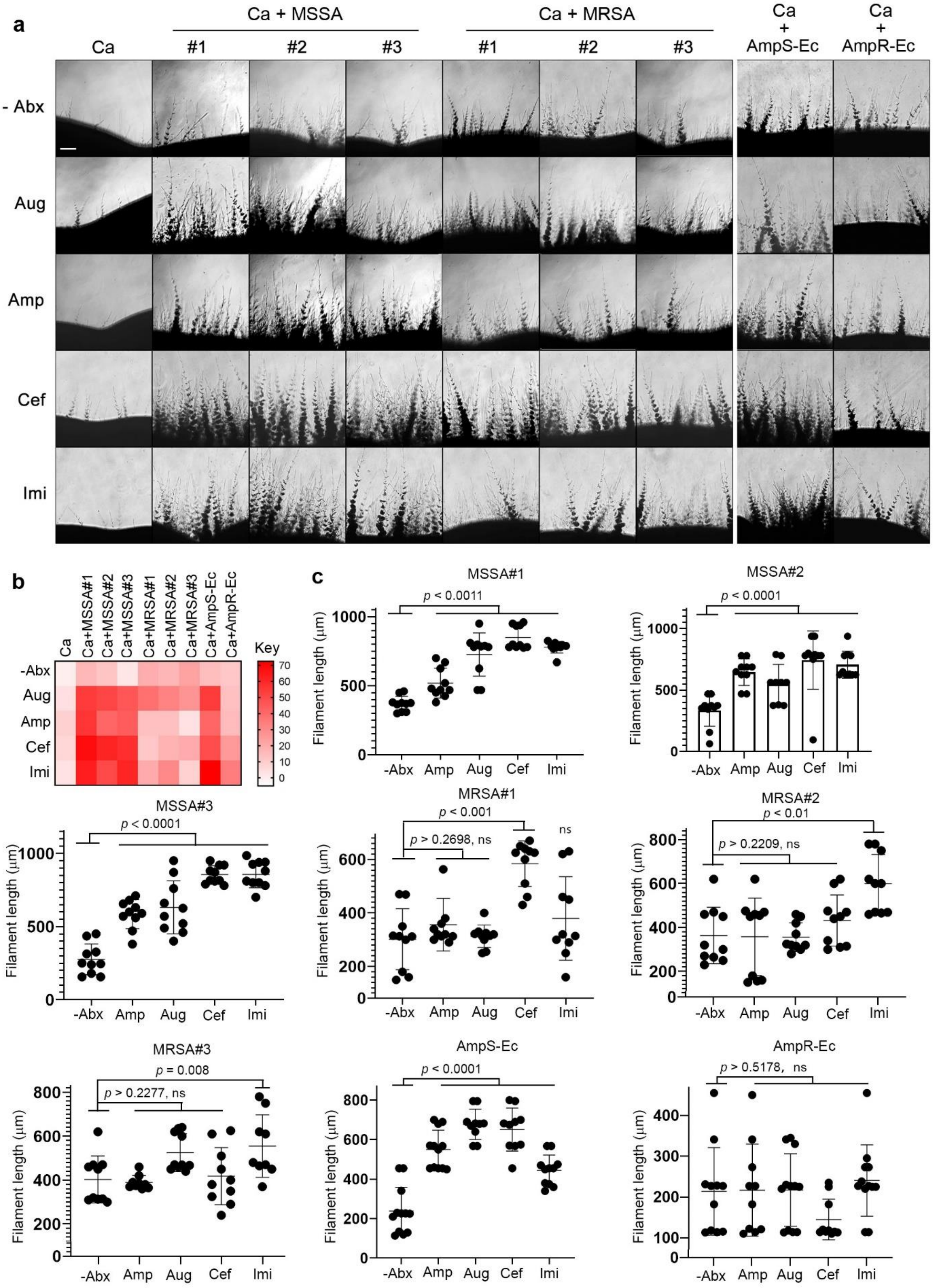

**Supplementary Figure 2** (related to Figure 2a of the main text). Resistant bacteria release significantly less PGN and hypha-inducing activity than sensitive strains in response to β-lactam antibiotics.

**a** *C. albicans* were grown side-by-side on LB plates with MSSA, MRSA, Amp-sensitive (AmpS-Ec) or resistant *E. coli* (AmpR-Ec) strains in the presence or absence of the indicated antibiotics at 30°C for 4 days as described in Figure 1a. Scale bar, 250 µm.

**b** Quantification of filamentous growth. The heatmap was generated as described in Figure 1c, which compares the degree of filamentous growth of the cultures shown in **a**.

**c** Average hyphal length of the cultures shown in **a**. Images of two representative areas of filamentous growth of each *C. albicans* patch were analyzed, and 10 filaments (n = 10) of each image were measured. P-values were determined using two-tailed unpaired t test. Bars are means ± SEM. ns, not significant.

Supplementary Figure 3

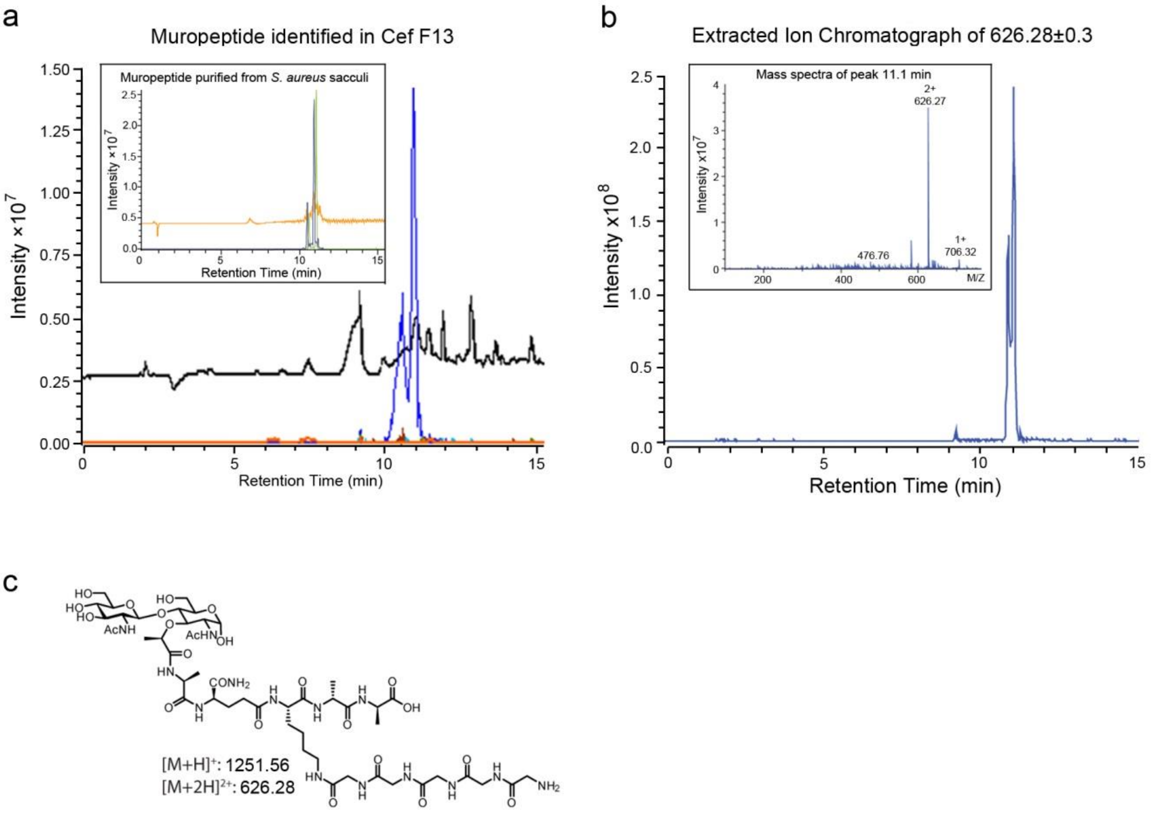


**Supplementary Figure 3** (related to Figure 2d and e of the main text). Identification of a muropeptide in the supernatant *S. aureus* culture treated with Cef or Amp.

To identify the active PGN subunits released after β-lactam antibiotic treatment of *S. aureus* cultures, HPLC fraction F13, which is descriebd in Figure 2d and e, were subjected to liquid chromatography-mass spectrometry (LC-MS) analysis. F13 of Amp and Cef-treated cultures showed similar UV and MS spectra (**a** and **b**), and the peaks were identified to have a molecular mass of 1251.56 $[M+H]^+$ and 628.26 $[M+H]^{2+}$ (**c**). The same muropeptide was also identified from purified *S. aureus* sacculi digested

with lysozyme (Supplementary Figure 4, compound e). The black line is TIC (total ion chromatogram), and the blue one is the EIC (Extract ion chromatogram).

**Supplementary Figure 4a. LCMS analyses of isolated muropeptides a-e.**

**compound a**

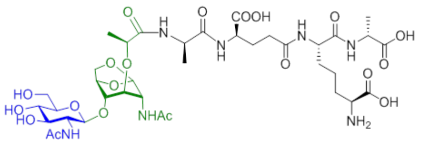


Chemical Formula: $C_{37}H_{59}N_7O_{20}$
Exact Mass: 921.38
[M+H]+: 922.39

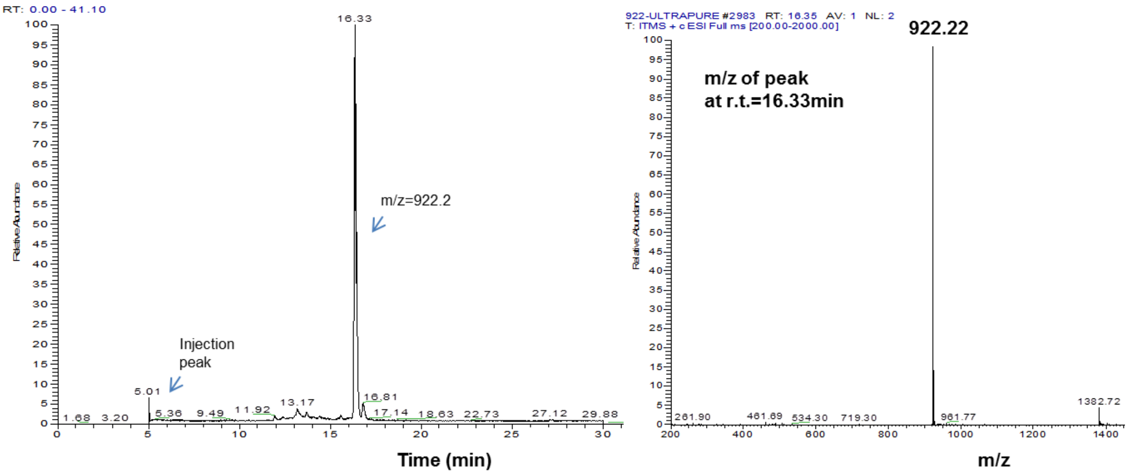


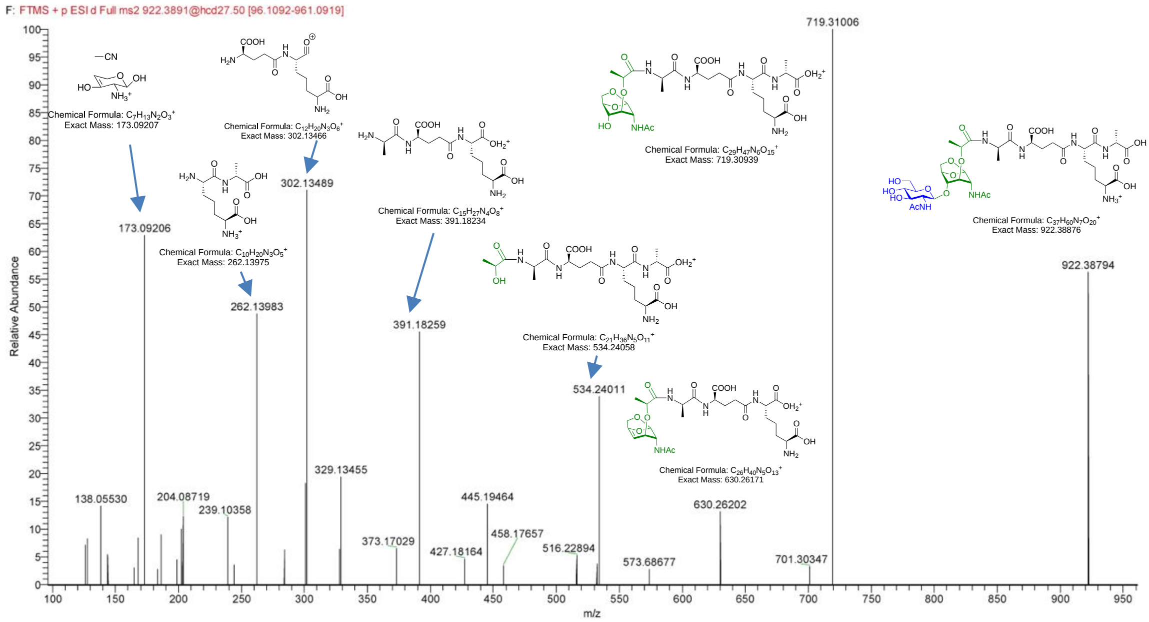

**compound b**

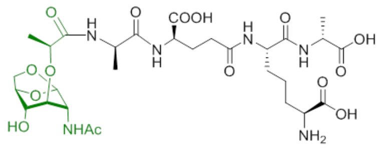


Chemical Formula: $C_{29}H_{46}N_6O_{15}$
Exact Mass: 718.30
[M+H]+: 719.31

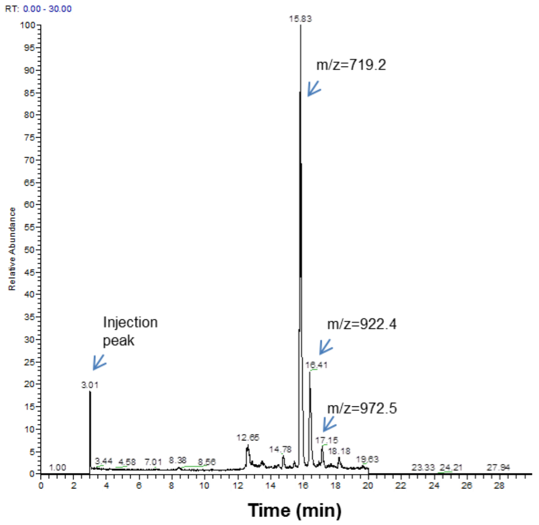


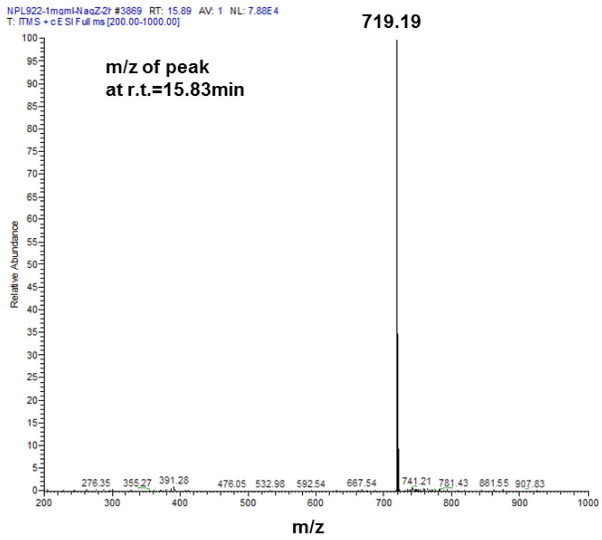


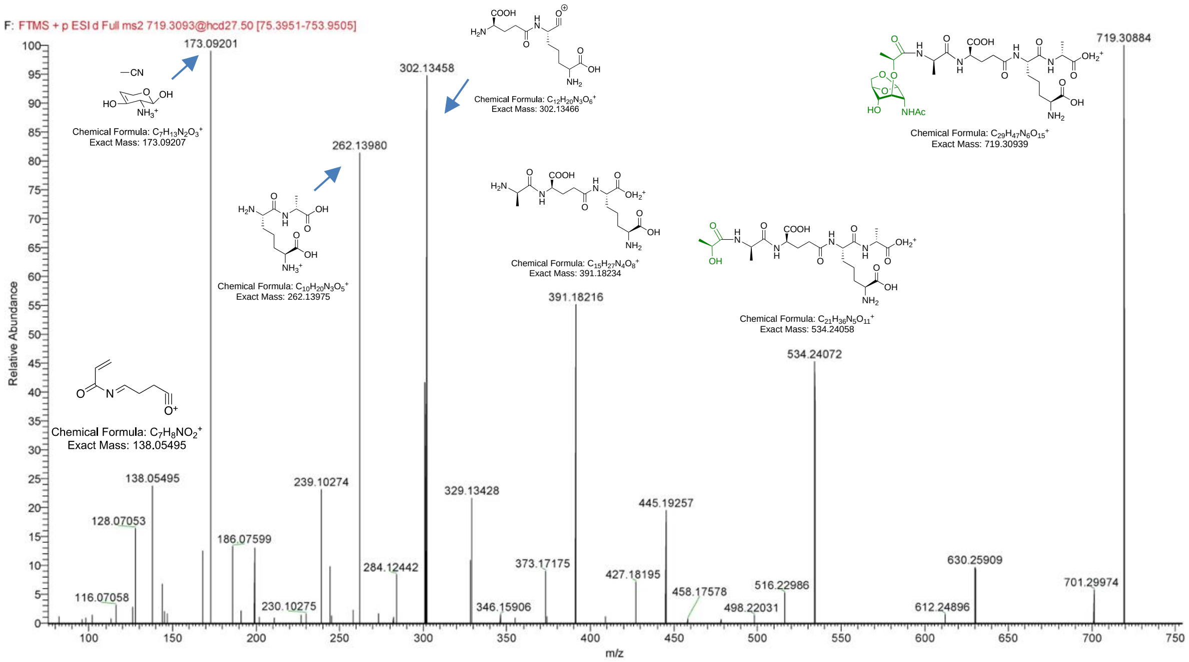

**compound c**

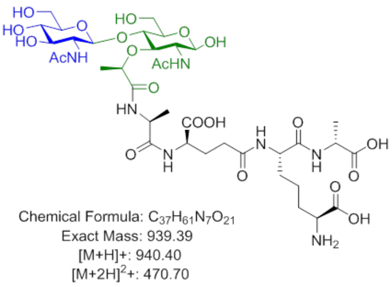

Chemical Formula: C37H61N7O21
Exact Mass: 939.39
[M+H]+: 940.40
[M+2H]2+: 470.70


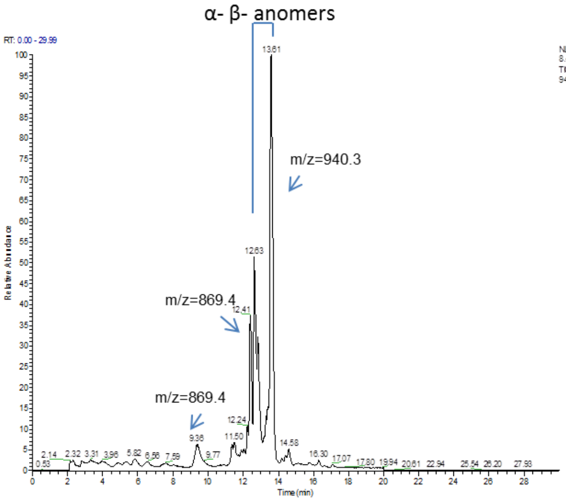

α- β- anomers
m/z=940.3
m/z=869.4
m/z=869.4
Relative Abundance
Time (min)


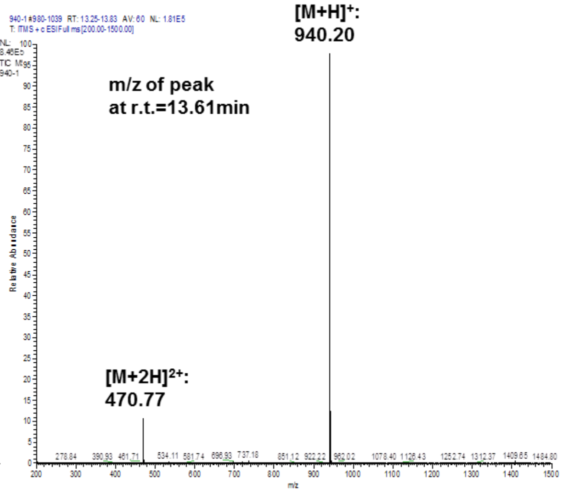

[M+H]+:
940.20
m/z of peak
at r.t.=13.61min
[M+2H]2+:
470.77
Relative Abundance
m/z


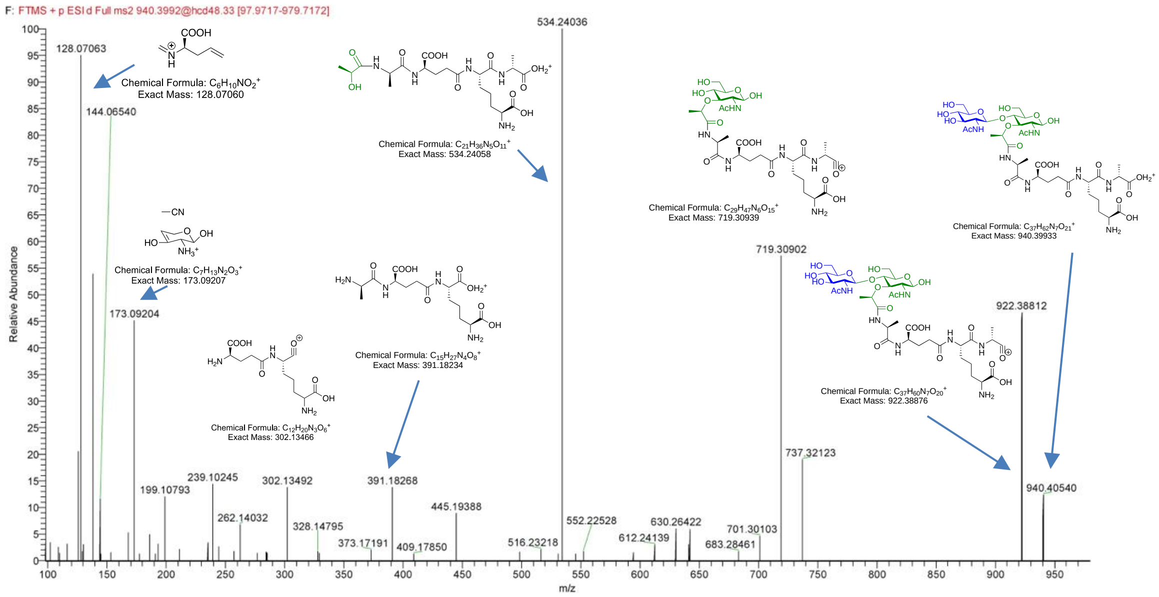

F: FTMS + p ESI d Full ms2 940.3992@hcd48.33 [97.9717-979.7172]
Relative Abundance
m/z
128.07063
Chemical Formula: C6H10NO2+
Exact Mass: 128.07060
144.06540
173.09204
Chemical Formula: C7H13N2O3+
Exact Mass: 173.09207
534.24036
Chemical Formula: C21H36N5O11+
Exact Mass: 534.24058
Chemical Formula: C29H47N6O15+
Exact Mass: 719.30939
719.30902
Chemical Formula: C37H62N7O21+
Exact Mass: 940.39933
922.38812
Chemical Formula: C37H60N7O20+
Exact Mass: 922.38876
940.40540
737.32123
Chemical Formula: C15H27N4O8+
Exact Mass: 391.18234
391.18268
Chemical Formula: C12H20N3O6+
Exact Mass: 302.13466
302.13492

## compound d

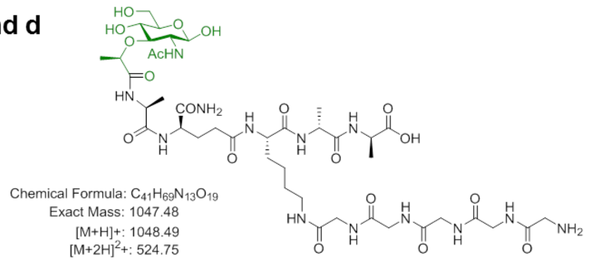

Chemical Formula: C41H69N13O19
Exact Mass: 1047.48
[M+H]+: 1048.49
[M+2H]2+: 524.75


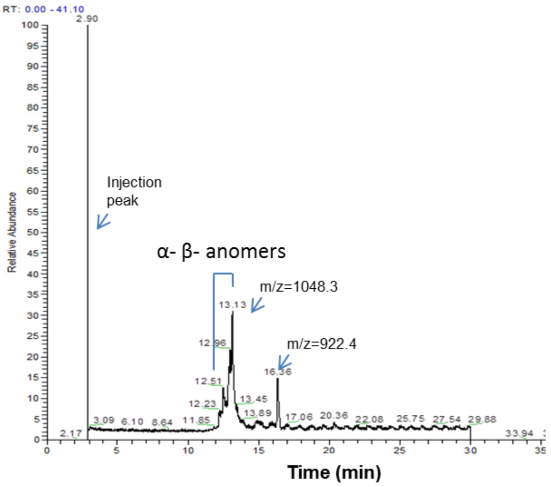

RT: 0.00 - 41.10
Injection peak
α- β- anomers
m/z=1048.3
m/z=922.4
Relative Abundance
Time (min)


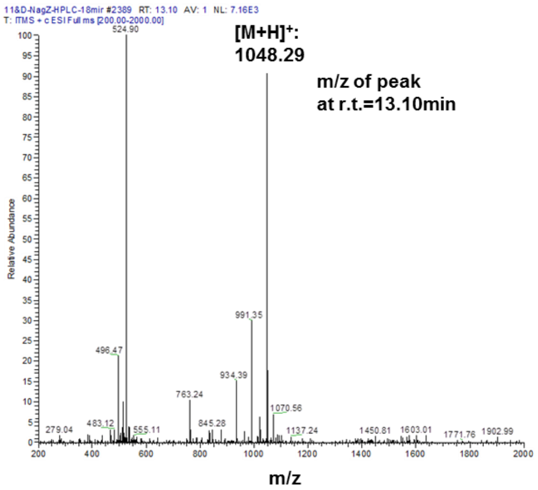

[M+H]+: 1048.29
m/z of peak at r.t.=13.10min
Relative Abundance
m/z


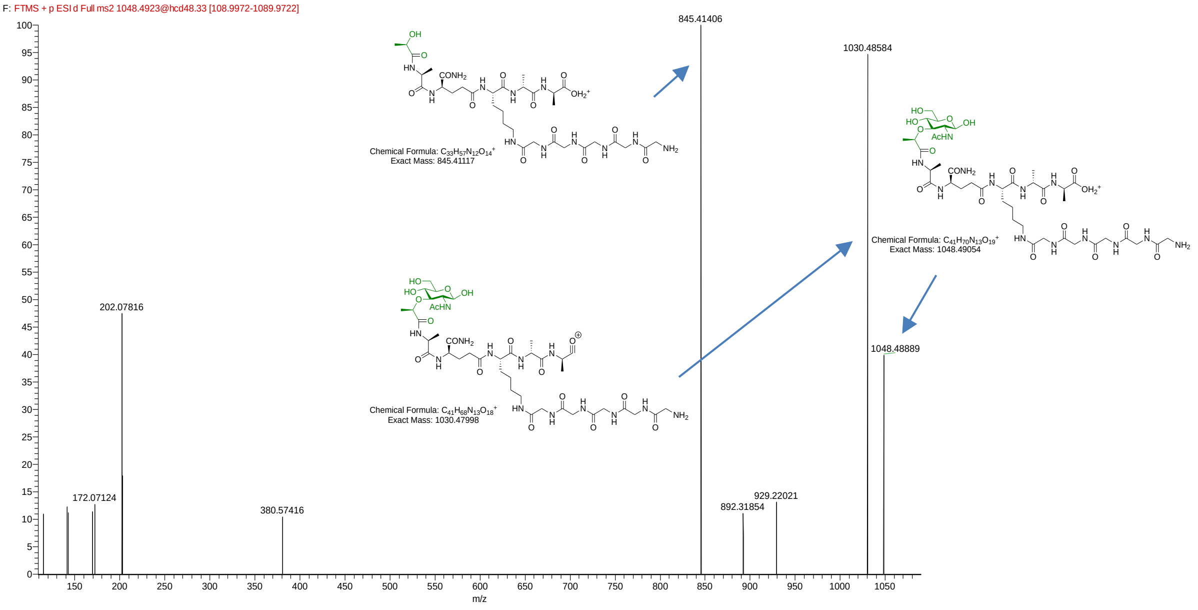

F: FTMS + p ESI d Full ms2 1048.4923@hcd48.33 [108.9972-1089.9722]
845.41406
1030.48584
1048.48889
202.07816
172.07124
380.57416
892.31854
929.22021
Chemical Formula: C33H57N12O14+
Exact Mass: 845.41117
Chemical Formula: C41H70N13O19+
Exact Mass: 1048.49054
Chemical Formula: C41H68N13O18+
Exact Mass: 1030.47998
m/z

**compound e**

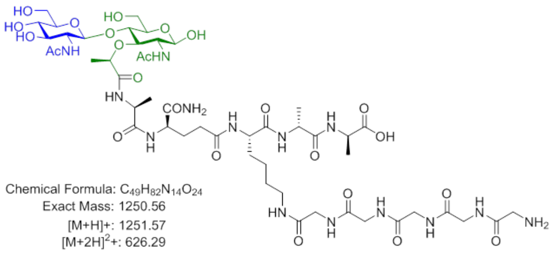

AcNH
AcHN
CONH2
OH
NH2
Chemical Formula: C49H82N14O24
Exact Mass: 1250.56
[M+H]+: 1251.57
[M+2H]2+: 626.29


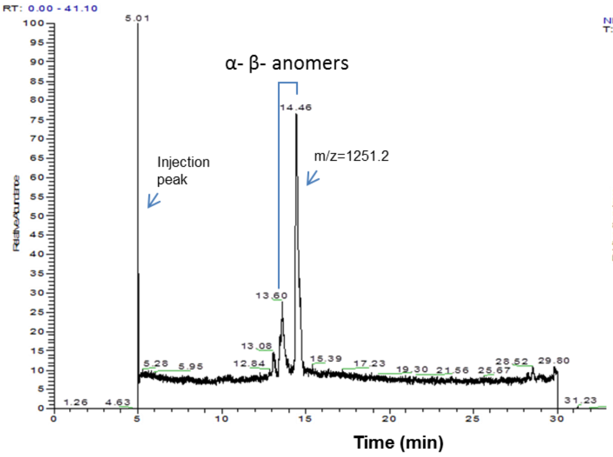

RT: 0.00 - 41.10
α- β- anomers
Injection peak
m/z=1251.2
5.01
14.46
13.60
13.08
12.84
5.28
5.95
15.39
17.23
19.30
21.56
25.67
28.52
29.80
31.23
1.26
4.63
Relative Abundance
Time (min)


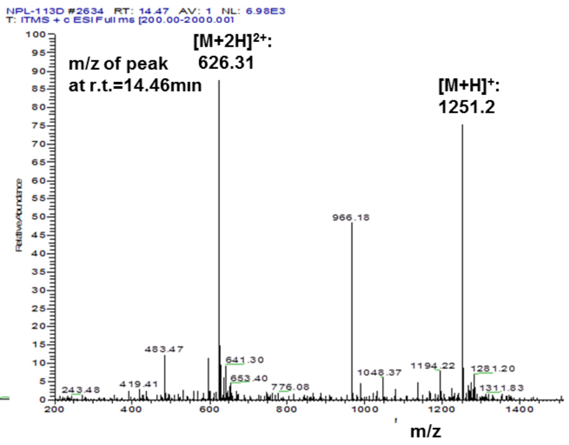

NPL-113D #2634 RT: 14.47 AV: 1 NL: 6.98E3
T: ITMS + c ESI Full ms [200.00-2000.00]
m/z of peak at r.t.=14.46min
[M+2H]2+: 626.31
[M+H]+: 1251.2
966.18
483.47
641.30
653.40
776.08
419.41
243.48
1048.37
1194.22
1281.20
1311.83
Relative Abundance
m/z


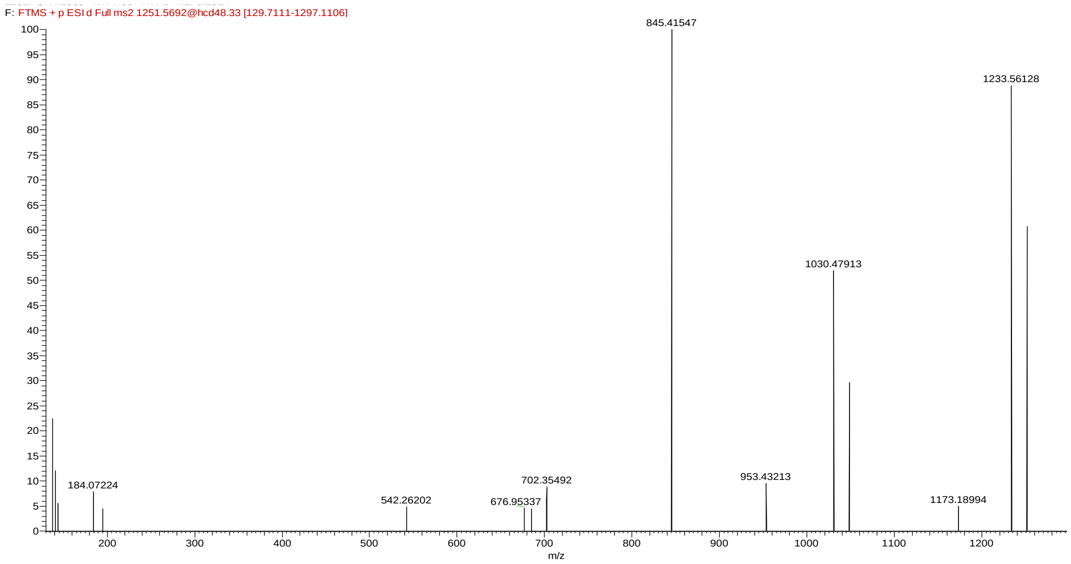

F: FTMS + p ESI d Full ms2 1251.5692@hcd48.33 [129.7111-1297.1106]
845.41547
1233.56128
1030.47913
953.43213
702.35492
676.95337
542.26202
184.07224
1173.18994
m/z

**Supplementary Figure 4b. Tabulation of hyphal induction by PGN subunits.**

| Compounds | Concentration (µM) | Hyphal Percentage (n = 100) | Hyphal Length (µm) mean±SD; (n = 100) |
|---|---|---|---|
| **MDP** | 500 | 2.4 | N.D. |
| | 1000 | 38.6 | 9.6±3.1 |
| | 2000 | 58.5 | 14.2±5.3 |
| **a** | 100 | 67.6 | 6.9±4.8 |
| | 200 | 83.8 | 12.0±3.1 |
| | 500 | 96.0 | 16.1±5.2 |
| **b** | 100 | 37.5 | 8.8±3.2 |
| | 200 | 63.5 | 10.5±3.4 |
| | 500 | 99.0 | 14.5±4.5 |
| **c** | 100 | 88.5 | 15.3±4.6 |
| | 200 | 100.0 | 18.3±5.4 |
| | 500 | 100.0 | 25.0±8.6 |
| **d** | 100 | 64.0 | 9.1±3.2 |
| | 200 | 87.0 | 12.0±4.2 |
| | 500 | 95.5 | 21.4±5.5 |
| **e** | 200 | 57.6 | 11.6±4.6 |
| | 500 | 76.9 | 12.9±3.5 |
| **Serum** | 5% | 100 | 26±6.6 |

**4c**

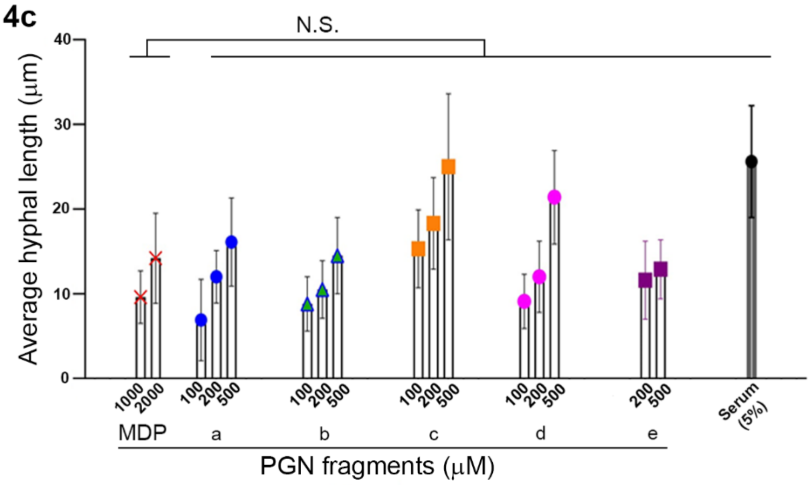


**4d**

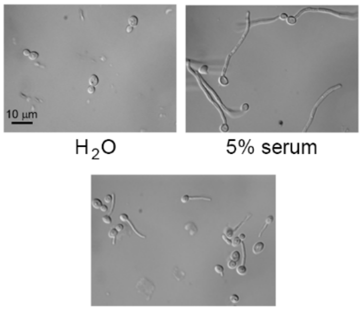


**Supplementary Figure 4** (related to Figure 4 of the main text).
**a**, LC/MS analysis of isolated muropeptides to confirm the purifty and structure identity. The six muropeptides described in Figure 3 were obtained by enzymatic digestion of isolated bacterial sacculi (*E. coli* for compound a-c; *S. aureus* for compound d-e), followed by HPLC purification. The identities and purifities of the purified compounds were confirmed by LC/MS. Left: total ion chromatogram (TIC) of the sample indicates the relative purity of each compound (identified by the major peak); right: mass spectra of the major peak in TIC which gives the desired m/z, confirming the identity of the compound.

LCMS analysis was perfomed using a Thermo accela LCQ Fleet LC-MS equipped with a C18 column (Waters symmetryshield RP18, 3.9mm × 150mm). The following gradient was used for the analysis: 100% A (Water + 0.1% formic acid) for 0-5min, followed with a linear gradient of 0-15% B (acetonitrile + 0.1% formic acid) for 5-20min. Run time between 2-20min was diverted to MS for analysis.

**b, c**, Tabulation of *C. albicans* hyphal growth induced by different muropeptides. Muropeptides were added to HBBS at the indicated concentrations, and hyphal induction was done at 37℃ for 2h. Fifty *C. albicans* cells (n = 50) in each test were analyzed to calculate the percentage and average length of hyphae. Fetal bovine serum (5%) was included as the positive control. Error bars, means±SD. Pair-wise comparison was made and significance was determined by two-tailed unpaired t test. ns, not signifcant.

**d**, Images of WT *C. albicans* cells induced with $H_2O$ (negative control), 5% serum (positive control), and MDP at 30℃ for 2 h. The experiment was repeated three times independently with similar results.

Supplementary Figure 5

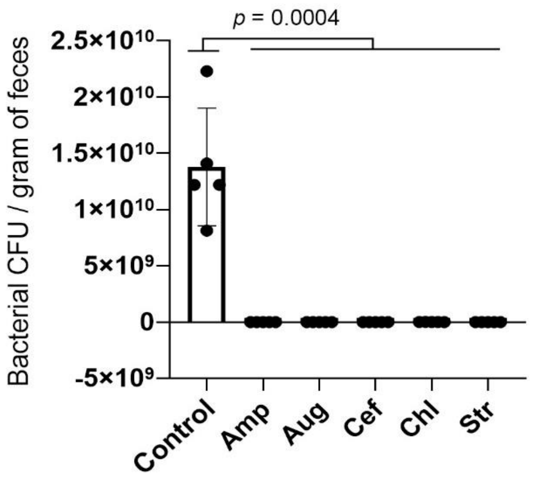


S**upplementary Figure 5** (related to Figure 4 of the main text). Test of the effectiveness of antibiotics on reducing the number of bacteria in the gut.
To minimize the inter-individual variations due to different drinking behaviors, each mouse (7-8 weeks old; n = 5) was orally administered 0.5 mL of an antibiotic solution (8 mg/mL for all antibiotics used) twice on day 1 with an 8 h interval. The drinking water also contained the same antibiotic. At 24 h of the antibiotic treatment, fresh feces were collected, weighed, resuspended in sterile PBS to a final concentration of 1 g/mL, and serially diluted 10-fold before spreading onto LB plates for incubation at 37°C for one day. Then, the CFUs on plates were counted. Three mice were used for each antibiotic treatment. P-values were calculated using two-tailed unpaired t test. Error bars: means±SEM.

Supplementary Figure 6

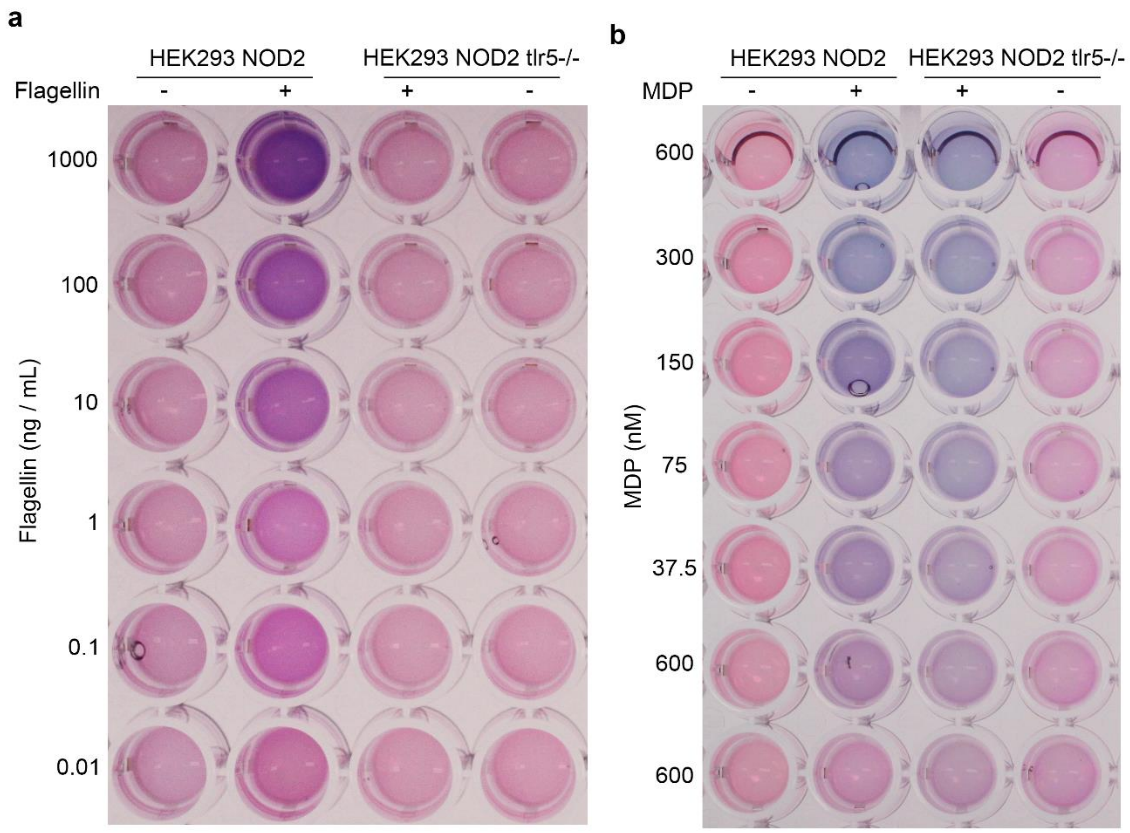


**Supplementary Figure 6.**

**a** HEK293 NOD2 tlr5-/- cells do not respond to flagellin.

The response of HEK293 NOD2 and HEK293 NOD2 tlr5-/- cells to flagellin was determined as described in Materials and Methods by replacing MDP with flagellin. Flagellin was purchased from Abcam (Catalog No. ab201366).

**b** HEK293 NOD2 tlr5-/- cells respond to MDP normally.

Supplementary Figure 7

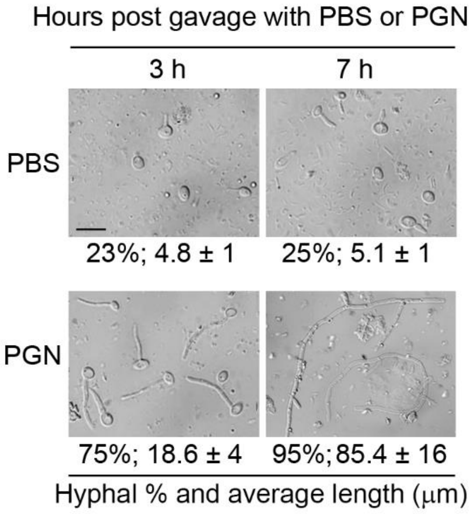


**Supplementary Figure 7.** Oral administration of mice with PGN caused *C. albicans* hyphal growth in the gut.

Balb/c mice (7-8 weeks old; n = 3) were administered orally with 0.5 mL of streptomycin solution (8 mg/mL) twice per day on day 1 and given drinking water supplemented with streptomycin at the same concentration throughout the experiment. After 4 days of streptomycin treatment, each mouse was gavaged with 1 × $10^8$ *C. albicans* yeast cells. At 48 h, mice were gavaged with 0.5 mL of PGN solution (4 mg in 0.5 mL) or PBS. Feces were collected at 3 and 7 h to examine *C. albicans* morphology. The percentage of hyphae was calculated, and the hyphal length was measured (n = 50). PGN polymers were prepared from *S. aureus* and digested with lysozyme as described in Methods. Scale bar, 5μm.

Supplementary Figure 8

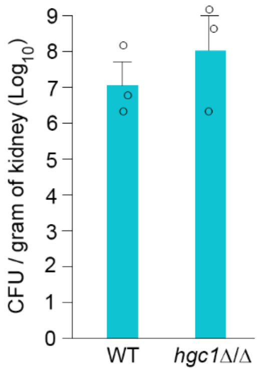


**Supplementary Figure 8.** WT *C. albicans* and the *hgc1*Δ/Δ mutant showed comparable ability to colonize mouse kidneys.

BALB/c mice (8-10 weeks old; n = 3) were used for this experiment. WT *C. albicans* (SC5314) and *hgc1*Δ/Δ cells (+*ARG4*+*HIS1*+*URA3*) were grown in YPD at 30 °C overnight before harvesting by centrifugation. Cells were resuspended in PBS at a density of 5 × $10^6$ cells / mL. Each mouse was inoculated via the tail vein with 200 μl of the cell suspension. At 48 h, all mice were sacrificed to harvest the kidney. The kidneys were homogenized in ice-cold PBS and 10-fold serially diluted before spreading aliquots onto YPD plates. CFU was counted after two days of incubation at 30 °C . Error bars, means ± SEM

Supplementary Figure 9

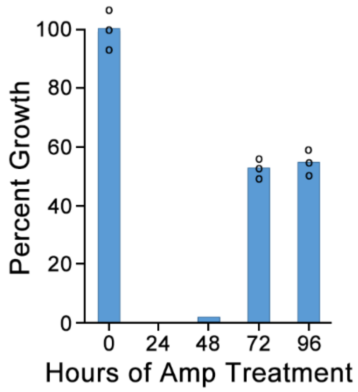


**Supplementary Figure 9.** Recovery of bacterial growth during Amp treatment.

Each mouse (n = 2) was orally administered 0.5 mL of Amp (8 mg/mL) twice on day 1 with an 8 h interval. The drinking water also contained Amp (4 mg/mL). At the indicated time points, fresh feces were collected, weighed, resuspended in sterile PBS to a final concentration of 1 g/mL, and serially diluted 10-fold before spreading onto LB plates for incubation at 37°C for one day. The CFUs on plates were counted. We only counted aerobic bacteria. The everage CFU at 0 h was treated as 100% and that at 24 h as 0. The experiment was repeated three times independently with similar results.

Supplementary Figure 10

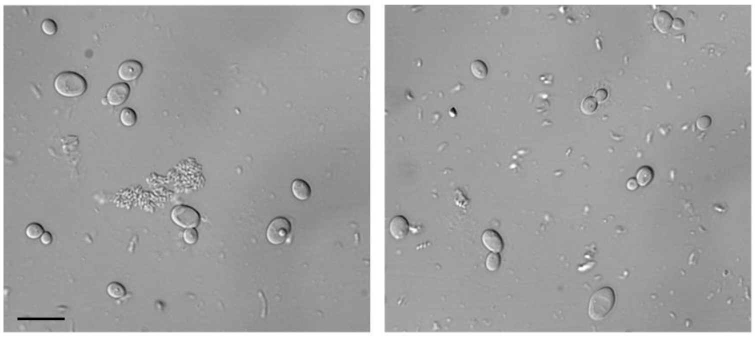

**Supplementary Figure 10.** Morphology of *C. albicans* in the feces of mice treated with the mixture of penicillin and streptomycin.

Three Balb/c female mice (7-8 weeks) were orally administered 0.5 mL of penicillin (2000 u/ mL)+streptomycin (2 mg/mL) solution twice on day 1. The same antibiotics were also added to the drinking water throughout the experiment, as described in Figure 4a. At 24 h, 1×108 wild-type *C. albicans* yeast cells were orally inoculated into mice. After 24 h, fresh feces were collected and resuspended in distilled water to examine *C. albicans* morphology. We detected only yeast cells, like in the feces of untreated mice (Figure 4a). The experiment was repeated three times independently with similar results.

**Table 1. *C. albicans* and bacterial strains used in this study.**

| Strains | Genotype | Source |
|---|---|---|
| SC5314 | Wild-type clinical isolate | |
| YZM147 | BWP17+*URA3+HIS1+ARG4+dTMT-SAT1* | Mamouei et al., 2017 |
| *hgc1*Δ/Δ | *hgc1*Δ::*ARG4*/*hgc1*Δ::*HIS1*, *URA3* | Zheng et al., 2004 |
| *Staphylococcus aureus* (MSSA#1) | Methicillin-sensitive | ATCC12598 |
| *Staphylococcus aureus* MRSA (MRSA#1) | Methicillin resistant | BAA1769 |
| *Staphylococcus aureus* (MRSA#2) | Clinical isolate, methicillin-resistant | National University Hospital, Singapore |
| *Staphylococcus aureus* (MRSA#3) | Clinical isolate, methicillin-resistant | National University Hospital, Singapore |
| *Staphylococcus aureus* (MSSA#2) | Clinical isolate, methicillin-sensitive | National University Hospital, Singapore |
| *Staphylococcus aureus* (MSSA#3) | Clinical isolate, methicillin-sensitive | National University Hospital, Singapore |
| *Streptococcus epidermidis* | Wild type | ATCC 35984 |
| *Streptococcus pyogenes* | Wild type | ATCC 12344 |
| *Escherichia coli* | Wild type | ATCC 12795 |
| *Pseudomonas aeruginosa* | Wild type | ATCC 14206 |
| AmpS-Ec (XL-1 Blue) | *recA1 endA1 gyrA96 thi-1 hsdR17 supE44 relA1 lac* [F´ *proAB lacI*q*Z*Δ*M15* Tn*10* (Tetr)] | Stratagene |
| AmpR-Ec | XL-1 blue cells were transformed with a plasmid carrying an AmpR gene | This study |

## References

Adikusuma F., Pfitzner C., and Thomas, P.Q (2017). Versatile single-step-assembly CRISPR/Cas9 vectors for dual gRNA expression. PLoS ONE 12(12): e0187236. https://doi.org/10.1371/journal.pone.0187236

Mamouei Z., Zeng G., Wang, Y.M., and Wang, Y. (2017). *Candida albicans* possess a highly versatile and dynamic high-affinity iron transport system important for its commensal-pathogenic lifestyle. *Mol Microbiol* 106(6), 986–998.

Zheng X., Wang, Y.M., and Wang, Y. (2004). Hgc1, a novel hypha-specific G1 cyclin-related protein regulates Candida albicans hyphal morphogenesis. *EMBO J* 23, 1845–1856.
