# Supplementary material for: A peptidoglycan storm caused by β-lactam antibiotic’s action on host microbiota drives Candida albicans infection
Source: Nat Commun. 2021 May 7;12:2560. doi: 10.1038/s41467-021-22845-2 (PMC8105390; doi:10.1038/s41467-021-22845-2)
Supplement: Supplementary file 2 — Reporting Summary [file 41467_2021_22845_MOESM2_ESM.pdf]

nature research

Corresponding author(s): Yue Wang

Last updated by author(s): Mar 23, 2021

# Reporting Summary

Nature Research wishes to improve the reproducibility of the work that we publish. This form provides structure for consistency and transparency in reporting. For further information on Nature Research policies, see Authors & Referees and the Editorial Policy Checklist .

## Statistics

For all statistical analyses, confirm that the following items are present in the figure legend, table legend, main text, or Methods section.

| n/a | Confirmed | |
|---|---|---|
| ☐ | ☒ | The exact sample size (*n*) for each experimental group/condition, given as a discrete number and unit of measurement |
| ☐ | ☒ | A statement on whether measurements were taken from distinct samples or whether the same sample was measured repeatedly |
| ☐ | ☒ | The statistical test(s) used AND whether they are one- or two-sided<br>*Only common tests should be described solely by name; describe more complex techniques in the Methods section.* |
| ☒ | ☐ | A description of all covariates tested |
| ☒ | ☐ | A description of any assumptions or corrections, such as tests of normality and adjustment for multiple comparisons |
| ☐ | ☒ | A full description of the statistical parameters including central tendency (e.g. means) or other basic estimates (e.g. regression coefficient) AND variation (e.g. standard deviation) or associated estimates of uncertainty (e.g. confidence intervals) |
| ☐ | ☒ | For null hypothesis testing, the test statistic (e.g. *F*, *t*, *r*) with confidence intervals, effect sizes, degrees of freedom and *P* value noted<br>*Give P values as exact values whenever suitable.* |
| ☒ | ☐ | For Bayesian analysis, information on the choice of priors and Markov chain Monte Carlo settings |
| ☒ | ☐ | For hierarchical and complex designs, identification of the appropriate level for tests and full reporting of outcomes |
| ☒ | ☐ | Estimates of effect sizes (e.g. Cohen's *d*, Pearson's *r*), indicating how they were calculated |

*Our web collection on statistics for biologists contains articles on many of the points above.*

## Software and code

Policy information about availability of computer code

| | |
|---|---|
| Data collection | ImageJ and Metamorph |
| Data analysis | Graph Pad Prism Software Version 4.00 and ImageJ |

For manuscripts utilizing custom algorithms or software that are central to the research but not yet described in published literature, software must be made available to editors/reviewers. We strongly encourage code deposition in a community repository (e.g. GitHub). See the Nature Research guidelines for submitting code & software for further information.

## Data

Policy information about availability of data

All manuscripts must include a data availability statement. This statement should provide the following information, where applicable:

- Accession codes, unique identifiers, or web links for publicly available datasets
- A list of figures that have associated raw data
- A description of any restrictions on data availability

All data that support the findings of this study are either included in this published article and its Supplementary information or available from the corresponding author upon request.

# Field-specific reporting

Please select the one below that is the best fit for your research. If you are not sure, read the appropriate sections before making your selection.

☒ Life sciences ☐ Behavioural & social sciences ☐ Ecological, evolutionary & environmental sciences

# Life sciences study design

All studies must disclose on these points even when the disclosure is negative.

| | |
|---|---|
| Sample size | The sample size for each experiment was determined based on pilot experiments, which provided an estimate of the effect size and of data variability. |
| Data exclusions | No data were excluded. |
| Replication | All in vitro and animal experiments were repeated at least three times independently over the past five years and done by independent researchers. All attempts at replication were successful and produced similar results. |
| Randomization | Mice of the same age and housed under the same conditions were allocated to treatment and control groups in a completely randomized manner without any bias. |
| Blinding | Critical experiments and data recording were repeated by technicians who had no knowledge of the reagents used and the possible outcome in relation to the hypothesis tested. |

# Reporting for specific materials, systems and methods

We require information from authors about some types of materials, experimental systems and methods used in many studies. Here, indicate whether each material, system or method listed is relevant to your study. If you are not sure if a list item applies to your research, read the appropriate section before selecting a response.

## Materials & experimental systems

| n/a | Involved in the study |
|---|---|
| ☐ | ☒ Antibodies |
| ☐ | ☒ Eukaryotic cell lines |
| ☒ | ☐ Palaeontology |
| ☐ | ☒ Animals and other organisms |
| ☒ | ☐ Human research participants |
| ☒ | ☐ Clinical data |

## Methods

| n/a | Involved in the study |
|---|---|
| ☒ | ☐ ChIP-seq |
| ☒ | ☐ Flow cytometry |
| ☒ | ☐ MRI-based neuroimaging |

## Antibodies

| | |
|---|---|
| Antibodies used | The 2E7 monoclonal antibody was generated in our own laboratory. Horseradish peroxidase-linked antibody was purchased from GE Healthcare (Code: NXA931). |
| Validation | Validation of 2E7 has been published in Nature Microbiology v4, 766-773 (2019). |

## Eukaryotic cell lines

Policy information about cell lines

| | |
|---|---|
| Cell line source(s) | HEK-Blue NOD2 cell lines were purchased from InvivoGen. HEK-Blue NOD2 tlr5-/- cells were derived from HEK-Blue NOD2 by mutating the LTR5 gene using CRISPR-Cas9. |
| Authentication | HEK-Blue cell line was not authenticated after receiving from the supplier. |
| Mycoplasma contamination | The cell lines were not checked for contamination by mycoplasma. |
| Commonly misidentified lines (See ICLAC register) | No commonly misidentified lines were used in this study. |

## Animals and other organisms

Policy information about studies involving animals; ARRIVE guidelines recommended for reporting animal research

| | |
|---|---|
| Laboratory animals | Balb/c mice, both male and female. Housing conditions are described in Methods. |
| Wild animals | No wild animals were used in this study. |
| Field-collected samples | No field-collected samples were used in this study. |

| | |
|---|---|
| Ethics oversight | All animal experiments were performed following animal ethics guidelines and protocols approved by the Institutional Animal Care and Use Committee (IACUC) of the Agency for Science, Technology and Research of Singapore. Other experiments were approved by the Institutional Biosafety Committee of the Institute of Molecular and Cell Biology, Singapore. |

Note that full information on the approval of the study protocol must also be provided in the manuscript.
